# Proton penetration mechanism and selective hydrogen isotope separation through two-dimensional biphenylene†

**DOI:** 10.1039/d3ra02993j

**Published:** 2023-09-15

**Authors:** Jiahui Zhao, Changti Pan, Yue Zhang, Xiyu Li, Guozhen Zhang, Li Yang

**Affiliations:** a Institutes of Physical Science and Information Technology, Anhui University Hefei Anhui 230601 China yangli91@mail.ustc.edu.cn; b Hefei National Research Center for Physical Sciences at the Microscale, School of Chemistry and Materials Science, University of Science and Technology of China Hefei Anhui 230026 China xylizy@ustc.edu.cn gzzhang@ustc.edu.cn; c Helmholtz-Zentrum Dresden-Rossendorf Bautzner Landstrasse 400 Dresden 01328 Germany; d Theoretical Chemistry, Technische Universität Dresden Mommsenstr. 13 Dresden 01062 Germany

## Abstract

Hydrogen isotope separation is of prime significance in various scientific and industrial applications. Nevertheless, the existing technologies are often expensive and energy demanding. Two-dimensional carbon materials are regarded as promising candidates for cost-effective separation of different hydrogen isotopes. Herein, based on theoretical calculations, we have systematically investigated the proton penetration mechanism and the associated isotope separation behavior through two-dimensional biphenylene, a novel graphene allotrope. The unique non-uniform rings with different sizes in the biphenylene layer resemble the topological defects of graphene, serving as proton transmission channels. We found that a proton can readily pass through biphenylene with a low energy barrier in some specific patterns. Furthermore, large kinetic isotope effect ratios for proton–deuteron (13.58) and proton–triton (53.10) were observed in an aqueous environment. We thus conclude that biphenylene would be a potential carbon material used for hydrogen isotope separation. This subtle exploitation of the natural structural specificity of biphenylene provides new insight into the search for materials for hydrogen isotope separation.

## Abstract 2

The proton penetration mechanism and the associated isotope separation behavior through two-dimensional biphenylene were systematically investigated and presented.

## Graphical abstract

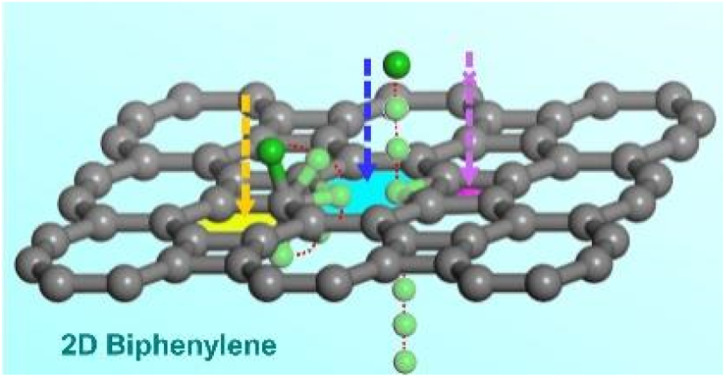

## Introduction

Selective separation of hydrogen isotopes plays a critical role in several nuclear and energy resource industries, for instance, in applications of tritium decontamination, as well as the fusion reactor deuterium-tritium fuel cycle.^1–5^ However, the current sieving materials often present a low separation factor and the existing approaches require complicated stages to achieve the desired separation requirement.^6^ These bottleneck problems lead to high operation costs and energy consumption,^7^ hindering large-scale utilization of hydrogen isotope separation and related technologies.^8^ To address these issues, an exploration for effective hydrogen isotope separation is pressing.

Since Geim *et al.* have found the proton permeability on monolayer two-dimensional (2D) materials such as graphene and hexagonal boron nitride,^6^ graphene-based systems have emerged as promising candidates for hydrogen isotope separation.^9,10^ As reported, previous experiments proposed a sandwiched graphene–Nafion membrane to separate proton and deuteron ions with a conductance ratio of 14.^11^ Vey recently, Yasuda *et al.* have fabricated graphene-based electrodes for hydrogen isotope enrichment, exhibiting a maximum proton–deuteron separation factor of around 25.^12^ Such superior performance can be fundamentally traced back to the specific physical and chemical feature of graphene-based materials, including the porous configuration, the atomically thin thickness, the high structural stability and mechanical flexibility.^13–22^

Furthermore, the emergence of transportation channels involving the naturally occurring topological Stone–Wales (55–77) defects, the vacancy defects of octagonal and pentagonal carbon rings can facilitate the proton penetration and hydrogen isotope separation.^23,24^ For instance, the Stone–Wales (55–77) defects are found to reduce the proton penetration barrier and exhibit a proton–deuteron selectivity of about 7.^24^ Previous studies also demonstrated the hydrogen isotope effect with observable proton–deuteron current difference on the defect graphene-based catalysts.^25^ Nevertheless, the concentration of naturally inevitable defects is relatively low, which are far from dominating the hydrogen-isotope separation process. Meanwhile, to increase the density of defects applicable for hydrogen isotope separation, the experimental engineering procedure often comes up with the multiple trial and error attempts.^26^ Thus, the search of alternative materials for the feasibly enhanced hydrogen isotope separation are still desired.

**Fig. 1:**
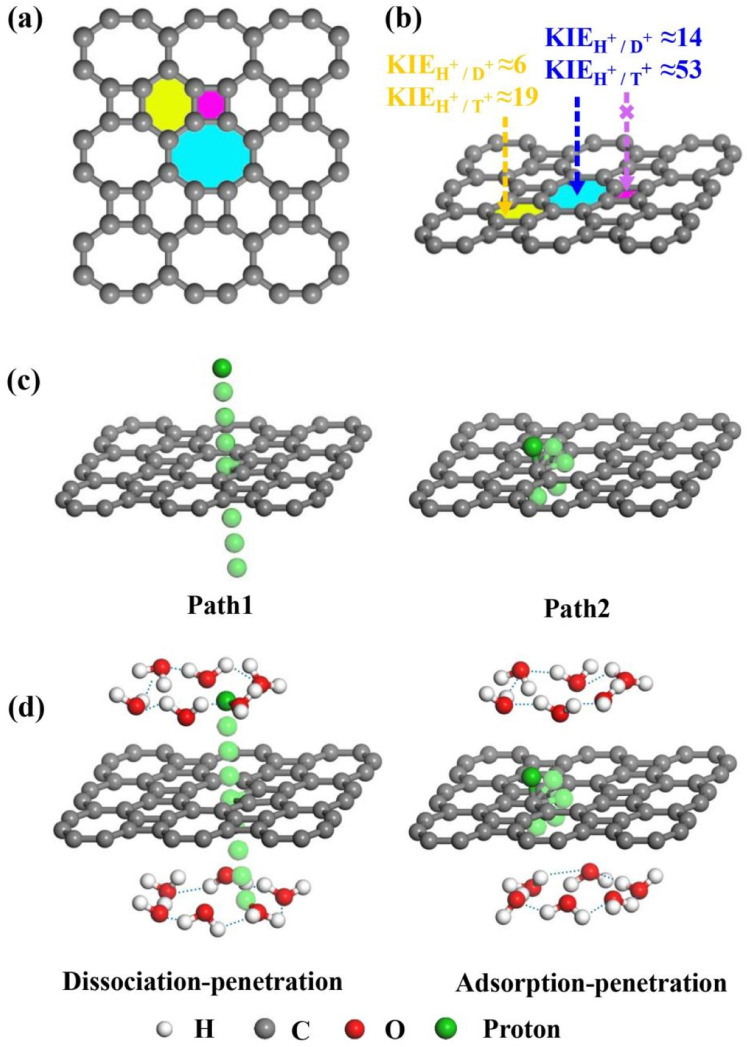
(a) Views of 2D biphenylene with multiple carbon rings. The purple, yellow, and blue areas separately represent the tetragonal, hexagonal, and octagonal carbon rings. (b) Schematic representation of proton (H^+^), deuteron (D^+^) and triton (T^+^) penetrating and separating through 2D biphenylene with different carbon ring sizes. In which, the KIE values correspond to the two lowest penetration barriers through hexagonal and octagonal carbon rings. (c) Two penetration modes through the 2D biphenylene layer in vacuum environment. (d) The profiles of proton dissociation–penetration and adsorption–penetration *via* the 2D biphenylene layer in aqueous solution.

Based on the hypothesis that defects promote separation, 2D biphenylene with carbon rings of different sizes may provide a potential approach for hydrogen isotope separation. As a novel allotrope of graphene, it has been synthesized *via* the interpolymer dehydrofluorination reaction consists of adjacent tetragonal, hexagonal, and octagonal carbon rings (Fig. 1a).^27,28^ Comparing to graphene, the intrinsic non-uniform biphenylene is equipped with three types of carbon rings, which may be treated as periodically topological defects (non-hexagonal rings) of graphene. Such enormous defects could probably present distinct penetration behavior for hydrogen isotope ions, thus offering a possible channel to separate them. If this scenario can be achievable, it will not only subtly take advantage of the natural structural specificity of biphenylene, but further, avoiding the difficulty and complexity of elaborate defect engineering for hydrogen isotope separation.^29^

## Results and discussions

Bearing this in mind, herein, we performed density functional theory calculations to illustrate the proton penetration mechanism and the associated hydron isotope separation behaviors through 2D biphenylene membranes (Fig. 1b). To gain a clear understanding on whether the aqueous solution could make sense upon the proton transmission, the penetration in both vacuum and aqueous condition have been illustrated in our work. Wherein, as to vacuum situation, the initial and final state in path 1 are protons physically adsorbed above the center of carbon rings on 2D biphenylene; while path 2 comes with the chemically adsorbed protons bonding with carbon atoms (Fig. 1c). In aqueous case, the dissociation–penetration route experiences with the initial proton originates from the dissociation of hydronium ion and return back to form another hydronium ion after penetration. Contrastively, protons in adsorption–penetration mode prefer to adsorb on the biphenylene layer and then flip over to the opposite side (Fig. 1d). The calculated proton transportation route through single-layer biphenylene in vacuum environment differs from the counterparts with explicit water molecules, suggesting that water environment should be considered in aqueous solution.^30^ Meanwhile, since the kinetic isotope effect (KIE) is normally used to estimate the efficiency of hydrogen isotope separation, the corresponding values have also been evaluated.^15,31^ In vacuum condition, proton could penetration through the hexagonal rings in path 1 mode with a low energy barrier of 0.89 eV and the associated KIE values are 5.87 and 19.17 for proton–deuteron and proton–triton systems, respectively. Furthermore, with a decreased penetration energy barrier of 0.63 eV, KIE values increase to 13.58 and 53.10 for the dissociation–penetration across the octagonal rings (Fig. 1b). Such high KIE rate ratios and low energy barriers indicate that 2D biphenylene with the unique configuration resembling the periodically topological-defect graphene could exhibit great potential for hydrogen isotope separation.

**Fig. 2:**
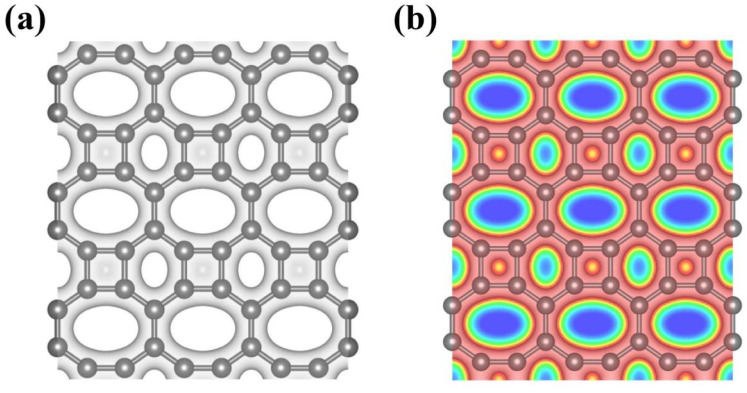
(a) Electron density clouds at an isosurface of 0.15 e^−^ bohr^−3^ for 2D biphenylene. The gray areas represent the electron clouds. (b) Two-dimensional charge density diagram of 2D biphenylene layer. The contour line in the charge density diagram represents 0.15 e^−^ bohr^−3^. The red and blue areas separately represent electron accumulation and depletion.

Previous studies have demonstrated that the proton penetration behavior is closely associated to the electronic feature of 2D crystals,^16^ therefore, to get an initial insight into the transmission mechanism, the electron density clouds and two-dimensional charge density diagram of biphenylene were plotted in Fig. 2. Unlike graphene, which exclusively consists of hexagonal carbon rings, the unequal ring size in 2D biphenylene suggests a particularly distinct distribution of electron density.^28^ A show in Fig. 2a, the tetragonal ring in biphenylene owns a quite dense electron density, which is filled of electron clouds in the pore. Such a compact distribution could be alleviated with the increase of ring size, as reflected by the decreased electron density of hexagonal and octagonal rings. This specific electronic structure can also be intuitively observed in Fig. 2b, showing more “porous” for the hexagonal and octagonal rings. Since dense electron clouds tend to prevent the facile proton penetration,^16^ it is reasonable to infer that proton is extremely difficult to transfer through the tetragonal rings. Contrastingly, the “porous” hexagonal and octagonal rings could provide the transmission channels. As a result, in the later simulation, we mainly focus on the proton penetration process across the hexagonal and octagonal carbon rings.

**Fig. 3:**
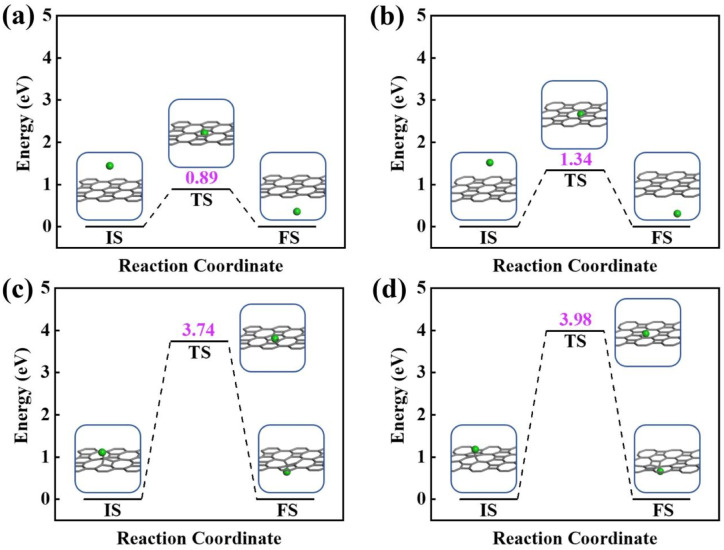
Energy profiles of proton conduction across 2D biphenylene materials in vacuum environment. Proton penetration through the hexagonal ring (a) and octagonal ring (b) in path 1. Proton transportation through the hexagonal ring (c) and octagonal ring (d) in path 2.

Next, the proton penetration mechanism on 2D biphenylene sheet were systematically investigated *via* the theoretical approaches. The transmission across biphenylene layer in vacuum environment were firstly computed as benchmark. Herein, we used the climbing image nudged elastic band (CI-NEB) simulation to identify the proton conduction pattern and the corresponding energy barriers.^32^ Hexagonal and octagonal rings of 2D biphenylene are considered as diffusion pores with two different modes. For the hexagonal rings in path 1, proton will first physically absorb on the free-standing biphenylene layer at a distance of about 3 Å (Fig. 3a). Then it will pass through the center of hexagonal ring *via* a nearly straight line perpendicular to the surface of the material. During this process, the total energy of the system increases until it reaches a maximum of transition state and then it falls down after passing through the hexagonal ring. In this scenario, the proton penetration energy barrier is calculated to be 0.89 eV, which suggests that proton could transfer across biphenylene layer. When it comes to the octagonal ring, proton experiences a bond formation and breakage with carbon atoms in octagonal ring rather than directly go through the ring center (Fig. 3b). This leads to an increased energy barrier of 1.34 eV, indicating that despite octagonal ring holds the most “porous” electron density, the local interaction between proton and carbon atoms in octagonal ring can affect the transportation process. The same route for proton passing through graphene layer was also simulated. The comparable values of 1.90 eV with previous reports not only demonstrates the reliability of our calculation,^16,29^ but also shows the reduction of conduction barrier through the topological-defect structure of biphenylene (Fig. S1†). Then, as to path 2 mode, both the hexagonal and octagonal ring exhibit strong chemical adsorption toward proton with the stable C–H bond. When starting from this chemisorbed configuration, one can easily found that the energy barriers are indeed very high with the values of 3.74 eV and 3.98 eV for hexagonal and octagonal rings, respectively (Fig. 3c and d). Such high energy barriers could be reasonably traced back to the continuous disruption of aromatic conjugation during the proton penetration process. Comparing to the physical absorption case in path 1, more energy is required to break the robust chemical bonds. As a result, proton can hardly pass through the biphenylene layer when it is chemically absorbed on the surface in path 2 pattern.

**Fig. 4:**
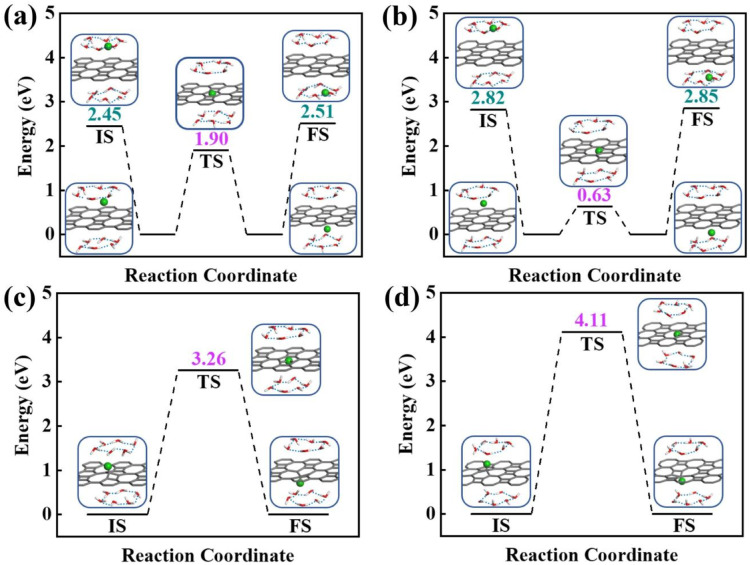
Energy profiles of proton passing across 2D biphenylene materials in aqueous environment. Proton penetration through the hexagonal ring (a) and octagonal ring (b) in the dissociation–penetration mode. The state between the initial and transition state is the dissociation configuration of hydronium ion. Proton transfer through the hexagonal ring (c) and octagonal ring (d) in adsorption–penetration route.

To obtain a clear understanding on whether the presence of aqueous environment will affect the proton penetration behavior, we further performed the proton transferring across 2D biphenylene with the incorporation of explicit water molecules. As to the dissociation–penetration mode, owing to the experimental observation that protons in aqueous environment will exist in the form of hydronium ion,^23^ the initial state is constructed by randomly combine a water molecule near the surface with proton to form hydronium ion. Then the proton dissociates from the hydronium ion to pass through the biphenylene sheet and integrate with a water molecule in the other side to form another hydronium ion (Fig. 4a and b). Specially, the dissociation of hydronium ion is energetically preferred, as reflected by the lower energy for the dissociated hydronium-ion state with respect to the preliminarily intact configuration. In this case, the proton penetration barrier through the hexagonal ring of biphenylene layer is 1.90 eV, almost 1 eV higher than that in vacuum environment. This increased penetration barrier from that in vacuum condition can be attributed to the deviation of proton transportation route, in which a bond formation and breakage process is observed rather than directly goes through the center of hexagonal ring, implying the considerable role of aqueous solution. Correspondingly, the energy barrier of proton conduction across the octagonal ring can also be tailored with the presence of water molecules, the significantly decreased value of 0.63 eV indicates that proton can readily go through the octagonal ring. It is known that the PBE functional tends to underestimate the barrier for proton transfer. Thus, some previously-reported density functionals have also been tested to simulate the proton penetration process.^15,24,33^ As shown in Fig. S2,† when employing the PBE0, B3LYP and optB88 functionals, the proton transmission pathways remain identical to that in PBE. Moreover, the energy barriers exhibit slight increases compared to the PBE value. These similar results suggest that the selection of density functional will not significantly affect the proton penetration mechanism, which is consistent with previous findings and reinforces the reliability of our calculations.^34^ Meanwhile, the akin transmission path of proton across the graphene layer was also computed, verifying the dependability of our simulation (Fig. S3†). While for the adsorption–penetration pattern, the proton transportation manner through 2D biphenylene is similar with those in vacuum environment (Fig. 4c and d). Within the strong interaction between proton and carbon atoms, the energy barriers for proton to penetrate across the hexagonal and octagonal rings retain high values of 3.26 eV and 4.11 eV, illustrating that proton is hard to pass through biphenylene layer in this mode.

Bader charge analysis was performed to obtain a deep insight into the chemical information about proton penetration mechanism. As depicted in Fig. S4 and S5,† during proton penetration, the carbon atoms of the hexagonal and octagonal rings in the biphenylene layer will donate charges to the approaching proton and subsequently reclaim them when the proton moves away from the layer. Notably, the charge migration reaches to the maximum values in the transition state, as evidenced by the charge variations of proton and the around carbon atoms, indicating that charge transfer may possibly contribute to the penetration energy barrier. A more intuitive picture of the charge displacement can be observed in the electron density clouds during proton transportation (Fig. S6 and S7†). When proton moves towards the hexagonal and octagonal rings, the electron density of the carbon rings decreases, such sparse distribution can be restored after penetration, which confirms the occurrence of charge donation and recollection during the transmission process.

**Fig. 5:**
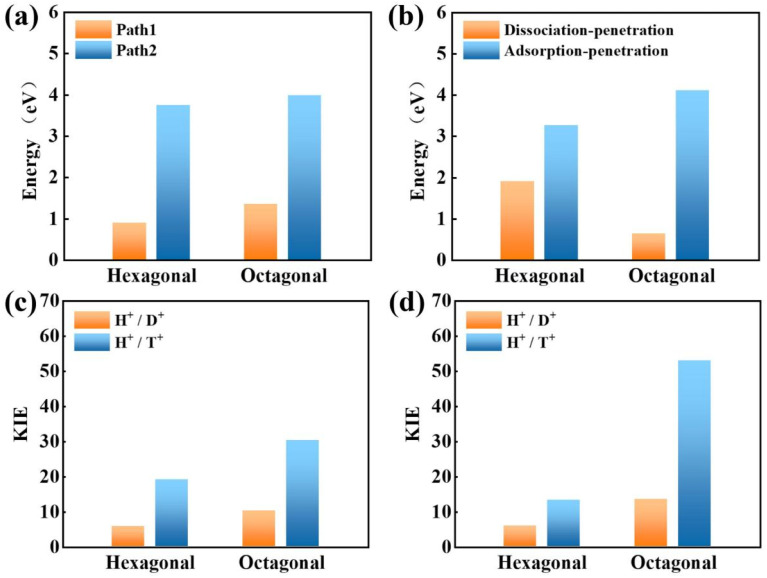
Proton penetration barriers through hexagonal and octagonal rings of biphenylene in vacuum (a) and aqueous (b) environment. Kinetic isotope effects of H^+^/D^+^ and H^+^/T^+^ for the proton transmission within path 1 mode in vacuum environment (c) and dissociation–penetration (d) pattern in aqueous environment.

Concerning the significant issue of hydrogen isotope separation, two fundamental factors should be clarified: the proton penetration energy barrier and the isotope separation efficiency. In which, a potential candidate for hydrogen isotope separation should simultaneously hold low penetration barrier to save the energy consumption and high isotope separation ratio for efficient sieving. Picking up all the above calculated proton penetration process and energy barriers of 2D biphenylene material, one can observe that proton passing through the hexagonal ring in path 1 (vacuum environment) and octagonal ring within the dissociation–penetration mode (aqueous environment) exhibit two lowest energy barriers (<1.0 eV, Fig. 5a and b). Although the specific transfer paths of these two patterns are distinct, they both possess the physiosorbed initial states either in the form of proton or hydronium ion. On the contrary, when the chemisorbed protons become the initial state, that are the cases in path 2 and adsorption–penetration mode, the strong interaction between proton and carbon atoms make it difficult to go through the biphenylene layer with the high energy barriers (>3.0 eV). This demonstrates that the weak binding of proton and biphenylene can facilitate the proton conduction process, conforming to the previous reports.^31^ Meanwhile, water molecules will make sense on the penetration mechanism, which can be rationally inferred from the discrepant transportation behavior in vacuum and aqueous environment.

When it comes to the hydrogen isotope separation efficiency, the kinetic isotope effect (KIE) rates were evaluated by using the vibrational frequencies of different initial and transition states. The KIE of proton transfer in path 2 and adsorption–penetration mode were not calculated for the high energy barriers that no protons could practically flow within these patterns. As to path 1 penetration in vacuum case, it can be seen that the hexagonal ring could exhibit comparable isotope effect with the KIE values of 5.87 and 19.17 for proton–deuteron (H^+^/D^+^) and proton–triton (H^+^/T^+^), respectively (Fig. 5c). Along with the low energy barrier of 0.89 eV, one can rationally speculated that when protons are physically absorbed above the hexagonal ring in vacuum environment, it can be separated from other hydron isotopes after penetration. Octagonal ring in this condition may probably contribute to the isotope separation with a larger KIE of 10.37 and 30.33, if the relatively higher transition barrier of 1.34 eV could be overcome. Considering the presence of aqueous solution, the KIE rate ratios of H^+^/D^+^ and H^+^/T^+^ for hexagonal rings in dissociation–penetration pattern were changed to 6.08 and 13.36, respectively (Fig. 5d). Nevertheless, hexagonal rings could not be regarded as an effective separation channel for the high penetration energy barrier (1.90 eV). Encouragingly, proton has superior isotope separation ratios with the significantly enhanced KIE values of 13.58 (H^+^/D^+^) and 53.10 (H^+^/T^+^) when passing across the octagonal rings. Within the low penetration barrier of 0.63 eV, this separation process could be reasonably expected, further confirming that 2D biphenylene can be used as an alternative candidate for hydrogen isotope separation. Meanwhile, the KIE rates for deuteron-triton (D^+^/T^+^) were also calculated, exhibiting lower values than the corresponding counterparts of H^+^/D^+^ and H^+^/T^+^ (Fig. S8 and Tables S1, S2†).

To clearly evaluate the effect of the aqueous environment on the penetration mechanism, systems with larger water clusters positioned above the 2D biphenylene layer were also investigated. Based on the previous literatures, equilibrated configuration possessing the lowest energy was extracted from the *ab initio* molecular dynamics (AIMD) simulations and identified for the further penetration calculations (Fig. S9†).^35–39^ In this extracted structure, the hydronium ion associated with proton penetration was involved in multiple hydrogen bonds. Then the proton transferring through the biphenylene sheet in the dissociation–penetration and adsorption–penetration routines were simulated. As illustrated in Fig. S10,† despite the initial water network differs from the prior structures with small water clusters, the proton penetration pathways and the corresponding energy barriers remained comparable. Considering the KIE rate ratios of H^+^/D^+^ and H^+^/T^+^, the values for hexagonal rings in dissociation–penetration mode were 5.33 and 12.57 respectively (Fig. S11†). Nevertheless, the relative high penetration energy barrier of 1.98 eV presents a challenge for efficient proton passage. While for the scenario of octagonal rings, proton could readily traverse to achieve satisfactory isotope separation with the high KIE values of 12.06 (H^+^/D^+^) and 48.23 (H^+^/T^+^). Although the calculated energy barriers and the KIE rate ratios may be influenced by the variations of water configurations and states, the qualitative depiction of proton penetration and isotope separation through the biphenylene layer remains consistent. This suggests that 2D biphenylene holds promise as a viable candidate for hydrogen isotope separation.

It should be noted that the nuclear tunneling effect need to be considered to comprehensively understand the proton penetration mechanism and the hydrogen isotope separation. Taking the proton transmission across the octagonal ring in dissociation–penetration mode as a demonstration, the minimum energy path along the proton transfer path was shown in Fig. S12.† The proton, deuteron and triton flow involving the quantum tunneling were evaluated *via* the Wentzel–Kramers–Brillouin (WKB) semiclassical approximation.^24,40^ As illustrated in Table S3,† the calculated tunneling for proton was approximately 10^−32^ (in Hartree atomic units of length/time), quite smaller than the classical flow (10^−15^). Deuteron and triton exhibit even lower values with heavier masses. Thus, quantum tunneling effects are negligible in the transportation process. The limited tunneling can be attributed to the wide reaction coordinate for proton penetration. In which, proton generated through the hydronium ion dissociation is relatively far away from the biphenylene layer due to the van der Waals interactions between the water molecules and the biphenylene sheet, causing the substantial decrease of the quantum tunneling participation. In this case, the corresponding selectivity for isotope separation is primarily dominated by classical particle flow, which is dependent on the mass deviations. In comparison to the large kinetic isotope effect of 13.58 (H^+^/D^+^) and 53.10 (H^+^/T^+^), the contribution of the isotope separation *via* nuclear tunneling is negligible, consistent with the prior reports.^15,34^ In addition, a similarly low tunneling contribution was observed in the scenario of proton penetration across the hexagonal ring. Taking proton traversing through the hexagonal ring along path 1 mode as a paradigm, the minimum potential energy path for proton penetration was presented in Fig. S13.† By applying the WKB approximation, the calculated quantum tunneling contribution for proton, deuteron, and triton were separately at the magnitude of 10^−28^, 10^−40^ and 10^−48^. Comparatively, these values are quite low in the transmission process when juxtaposed with their corresponding classical flow of 10^−19^ (Table S4†). Consequently, the dominant role of classical particle flow could still be maintained, leading to the mass-dependent isotope selectivity. Therefore, it is reasonable to speculate that the isotope separation during proton penetration is primarily governed by the relatively higher kinetic isotope effect.

## Conclusions

In conclusion, we have systemically investigated the proton penetration and isotope separation behaviors through two-dimensional biphenylene. The unique structure with three different types of carbon rings resembles the periodically topological-defect graphene, in which the “porous” hexagonal and octagonal rings can serve as the proton transportation channels. To clarify the transportation mechanism, the proton conduction in vacuum and aqueous environment separately within two distinct modes were demonstrated. Our simulations found that proton penetration through hexagonal rings in path 1 mode and across the octagonal rings in dissociation–penetration pattern could exhibit relatively low energy barriers, implying the feasibility of proton transmission. Furthermore, large KIE rate ratios of and for proton–deuteron (13.58) and proton–triton (53.10) *via* the dissociation–penetration through the octagonal rings have been observed. As such, superior isotope separation could be expected under this condition. Based on the knowledge of penetration barrier and KIE values, we conclude that 2D biphenylene is likely to be a promising two-dimensional carbon material for hydrogen isotope separation. This work improves the understanding of proton penetration and isotope separation manner, which is helpful to the search of potential materials as proton sieves.

## Computational details

### DFT calculations

Theoretical calculations were conducted by using the non-empirical Perdew–Burke–Ernzerhof (PBE) exchange-correlation functional implemented in the CP2K code.^41,42^ The cell sizes are 13.6 Å × 11.2 Å × 20.0 Å for two-dimensional (2D) biphenylene systems. Density functional theory (DFT)-D3 dispersion correction was adopted to consider the van-der-Waals interactions in the systems. The Qucikstep method was employed in our work, with the wave functions expanded to the localized bivalent polarized basis set.^43^ The kinetic energy cutoff was chosen to be 500 Ry. The minimum energy paths and energy barriers of proton penetration through 2D biphenylene were evaluated using the climbing-image nudged elastic band (CI-NEB) method.^44^ To obtain the reaction rates in simple harmonic approximation including the zero-point energy for the penetration process, the vibrational frequencies of the initial states and transition states were simulated by the finite-difference method.^19,34,45^

To gain a clear understanding of whether the presence of water can affect the proton penetration process, we employed a simplified aqueous model with a closed hydrogen network consisted of six water molecules on each side of the 2D biphenylene. Such simplified model was constructed because those water molecules around the penetration site could possibly influence the proton transmission behavior.^22^ With the addition of protons, this model enables us to demonstrate the proton transfer through the biphenylene layer either in the initial formation of hydronium ion or in the chemisorb state bonding with carbon atoms.

*Ab initio* molecular dynamics (AIMD) simulations was also performed to extract the equilibrated structures incorporating large water molecules. Water molecules were uniformly distributed in the vacuum space to approximately achieve the density of the water close to the experimental value of 1 g cm^−3^ under standard conditions.^22^ Then the systems were equilibrated in the canonical NVT ensemble for 10 ps with a time step of 1 fs, keeping a constant temperature of 300 K. As time evolves, the structures almost remain unchangeable with the energies fluctuating around the equilibrium state. The energy-minimized structure collected from the equilibrated trajectories was identified as the initial structure for the further penetration analysis.

### Kinetic isotope effect evaluation

In this work, the kinetic isotope effect was calculated to estimate the hydrogen isotope separation efficiency. According to Arrhenius equation, the ratio of kinetic isotope effect is given byKIE = *k*_1_/*k*_2_
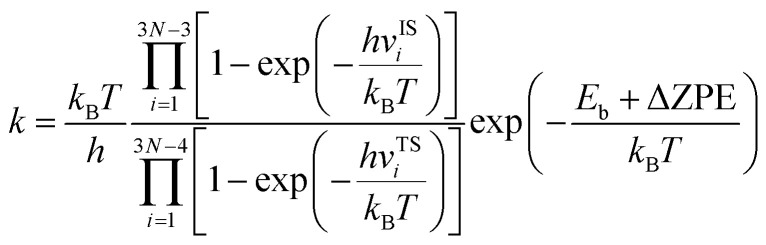
where *k*_1_ and *k*_2_ are the reaction rates of the two hydrogen isotope ions, *N* is the number of atoms in the system, *k*_B_ is the Boltzmann constant and *T* refer to the temperature in kelvin, respectively. *v*^IS^_i_ and *v*^TS^_i_ are the vibrational frequencies of the initial and transition states, respectively. In which, transition state represents the state with the highest potential energy along the lowest reaction energy profile. *E*_b_ is the energy barrier calculated by density functional theory. ΔZPE is the zero-point-energy correction with respect to the reaction potential which denotes as the zero-point energy difference between the transition state and the initial state, it can be calculated according to the following formula:^22,38^
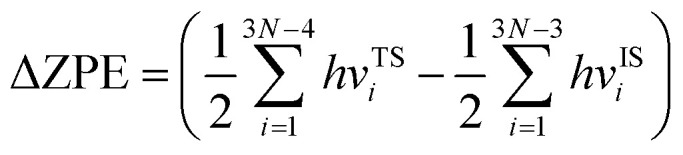


Quantum tunneling was estimated using the Wentzel–Kramers–Brillouin (WKB) approximation.^46^ In which, the transmission coefficient *T* for a one-dimensional energy barrier with the energy *E* can be calculated as:
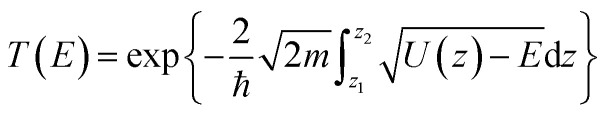
here, *z* is the coordinate along the minimum tunneling path, *z*_1_ and *z*_2_ are two positions where *U*(*z*_1_) = *U*(*z*_2_) = *E*. The energy *E* of the particle is smaller than the maximum barrier of *U*_max_, the over-barrier reflection was neglected by setting *T*(*E*) = 1 when *E* > *U*_max_.

By employing a one-dimensional transition-state model proposed in the work of Tkatchenko *et al.*,^40^ the particle flow through the barrier *via* quantum tunneling contribution can be obtained as (detailed code could be referred to the ESI†):
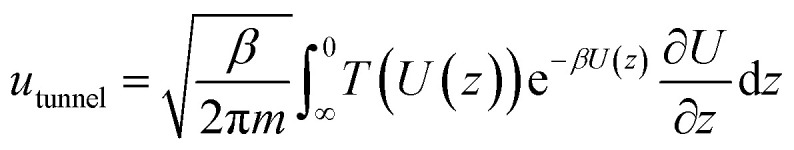


The classical particle flow is illustrated by:
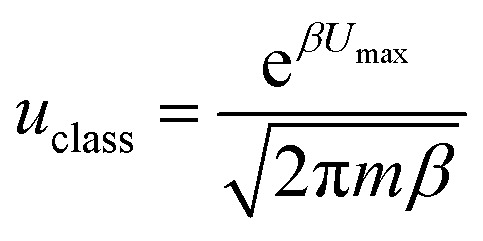

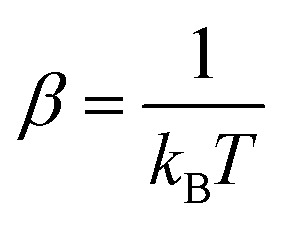
, *k*_B_ is the Boltzmann constant, combing the quantum tunnelling and classical flow together, the total particle flow could be described as:
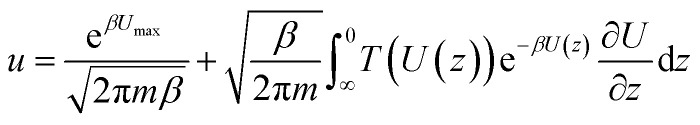


## Conflicts of interest

There are no conflicts to declare.

## Supplementary Material

RA-013-D3RA02993J-s001
