# Supplementary material for: Proton penetration mechanism and selective hydrogen isotope separation through two-dimensional biphenylene
Source: RSC Adv. 2023 Sep 15;13(39):27590–8. doi: 10.1039/d3ra02993j (PMC10503273; doi:10.1039/d3ra02993j)
Supplement: RA-013-D3RA02993J-s001 [file RA-013-D3RA02993J-s001.pdf]

Electronic Supplementary Material (ESI) for RSC Advances.
This journal is © The Royal Society of Chemistry 2023

Supporting Information

Proton penetration mechanism and selective hydrogen isotope separation through two-dimensional biphenylene

Jiahui Zhao,[a†] Changti Pan,[a†] Yue Zhang,[a] Xiyu Li,[*b] Guozhen Zhang,[*b] Li Yang [*a,c,d]

[a] *Institute of Physical Science and Information Technology, Anhui University, Hefei, Anhui 230601, China*

[b] *Hefei National Research Center for Physical Sciences at the Microscale, School of Chemistry and Materials Science, University of Science and Technology of China, Hefei, Anhui 230026, China*

[c] *Helmholtz-Zentrum Dresden-Rossendorf, Bautzner Landstrasse 400, Dresden 01328, Germany*

[d] *Theoretical Chemistry, Technische Universität Dresden, Mommsenstr. 13, Dresden 01062, Germany*

Corresponding Author: xylizy@ustc.edu.cn; gzzhang@ustc.edu.cn; yangli91@mail.ustc.edu.cn.

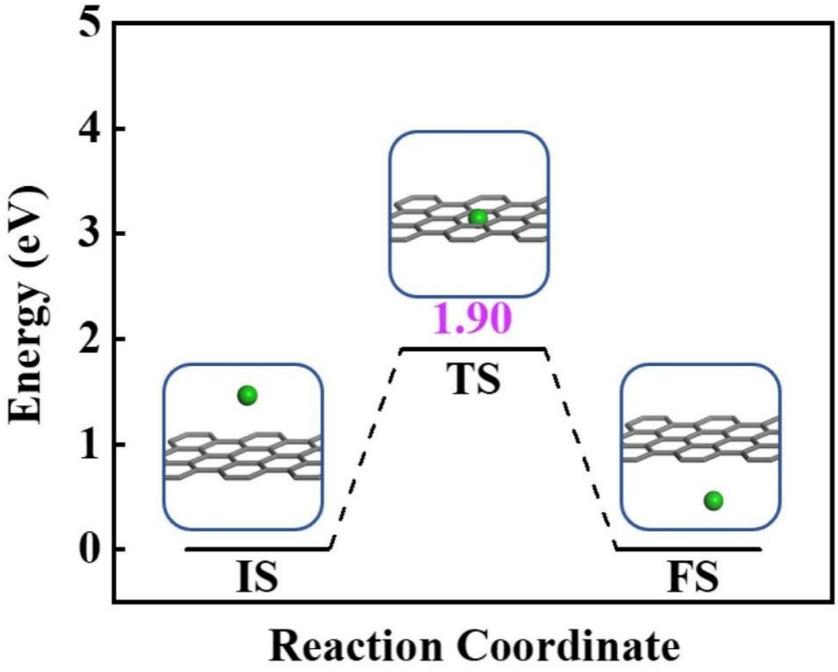

Fig. S1 Energy profiles of proton conduction across the graphene layer in vacuum environment within the path 1 mode. The gray, white and green spheres represent C and H atoms and the penetrated proton, respectively.

We considered the penetration of the proton through graphene in vacuum environment within path 1 pattern for comparison. As illustrated above, proton physically absorbed onto graphene at a distance of 3 Å, which resembles that of 2D biphenylene. CI-NEB simulation found that proton will penetrate through the center of hexagonal ring via a nearly straight line perpendicular to the surface of the graphene. The proton penetration barrier is calculated to be about 1.90 eV, which is consistent with the previous reports[1], demonstrating the reliability of our simulations for the corresponding 2D biphenylene.

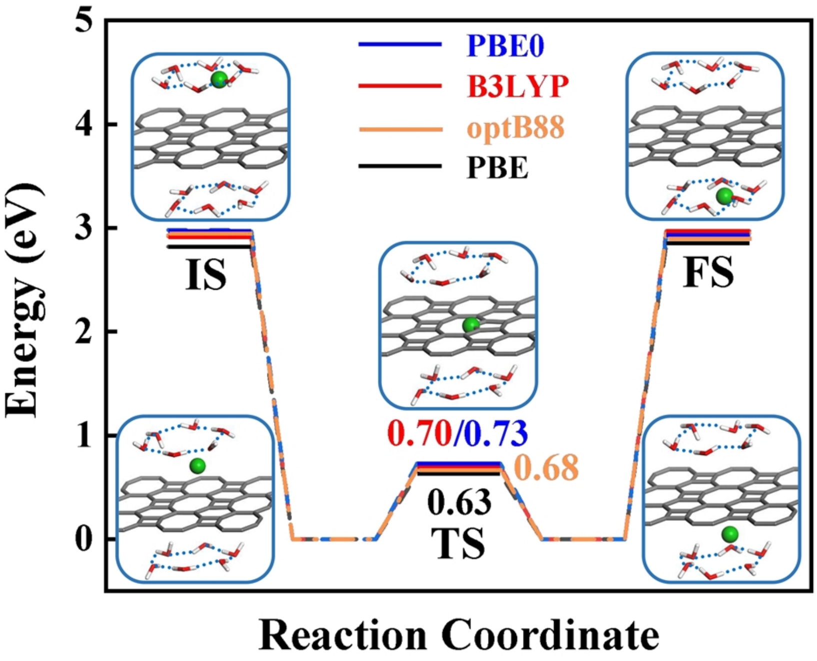

Fig. S2 Energy profiles of proton penetration across the octagonal ring within the dissociation-penetration mode under different density functionals.

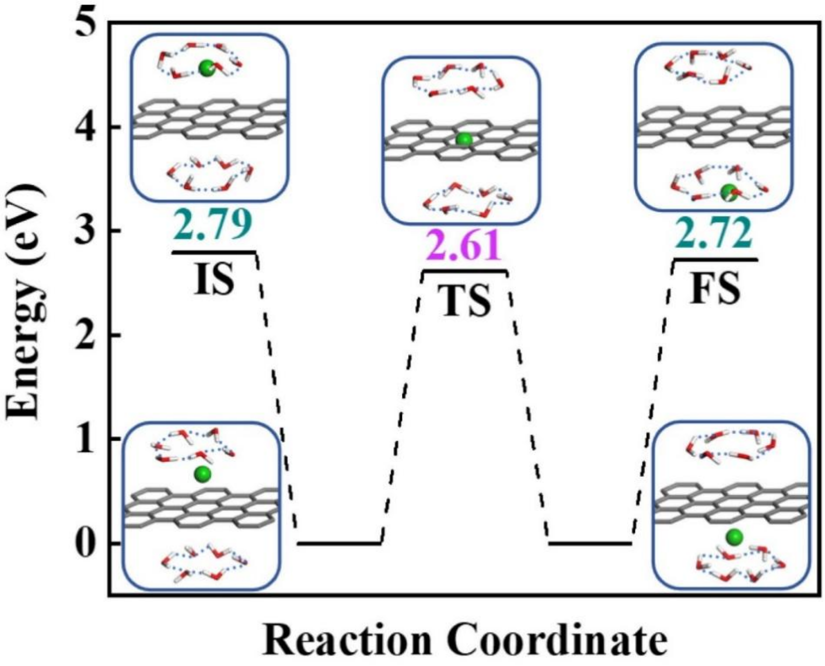

Fig. S3 Energy profiles of proton transfer through the graphene layer in aqueous environment within the dissociation-penetration pattern. The gray, red, white and green spheres represent C, O, H atoms and the penetrated proton, respectively.

The transmission process of proton across graphene in aqueous environment within dissociation-penetration mode is simulated for comparison. Similar with 2D biphenylene, the whole penetration process is: proton firstly dissociates from a hydronium ion, penetrates through graphene and then integrates with a water molecule in the other side to form another hydronium ion. The calculated energy barrier is about 2.61 eV, almost in line with the previous literatures on graphene[2], further verifying the dependability of our simulation on 2D biphenylene.

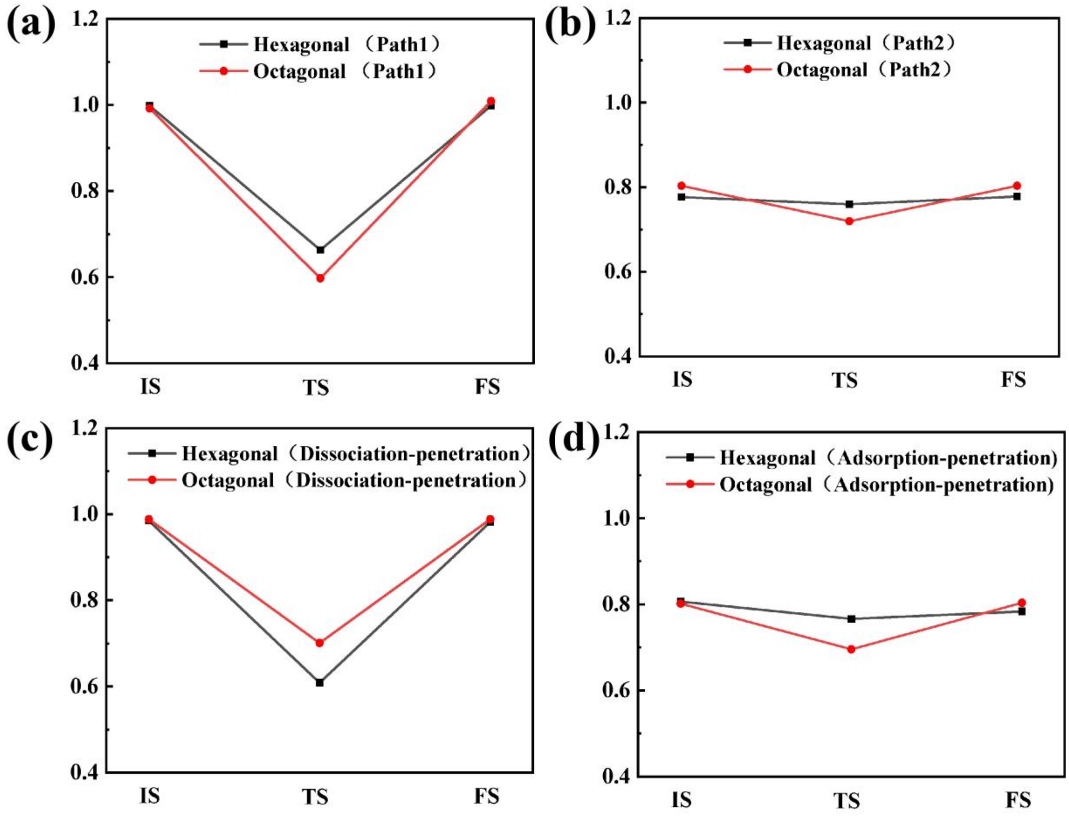

Fig. S4 Charge variations of proton transmission through biphenylene layer under different environments. Bader charge analyses of the proton in path 1 (a), path 2 (b), dissociation-penetration (c) and adsorption-penetration route (d).

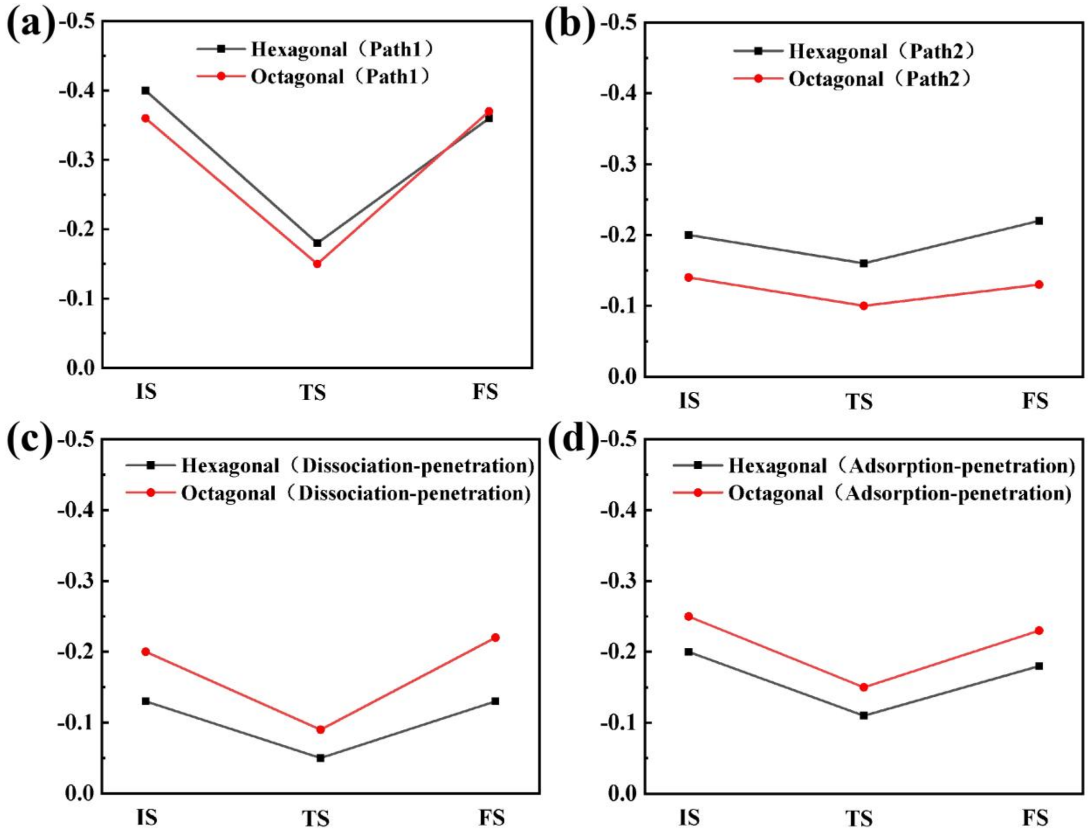

Fig. S5 Charge variations of carbon atoms in the hexagonal and octagonal rings during proton transmission through biphenylene layer under different environments. Bader charge analyses on hexagonal ring and octagonal ring in path 1 (a), path 2 (b), dissociation-penetration (c) and adsorption-penetration route (d).

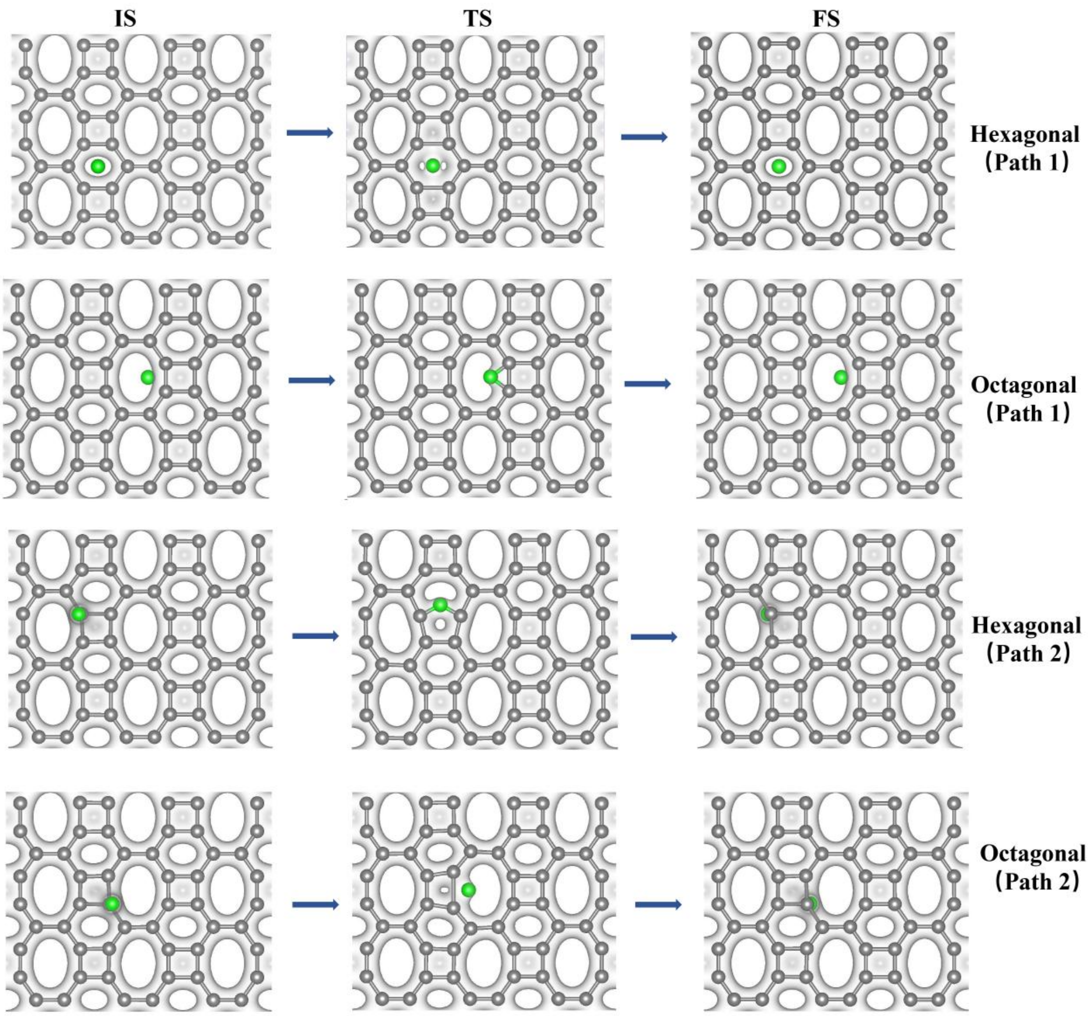

Fig. S6 Electron density clouds at an isosurface of 0.15 e$^-$/Bohr^3 for proton penetration through the biphenylene layer in vacuum environment.

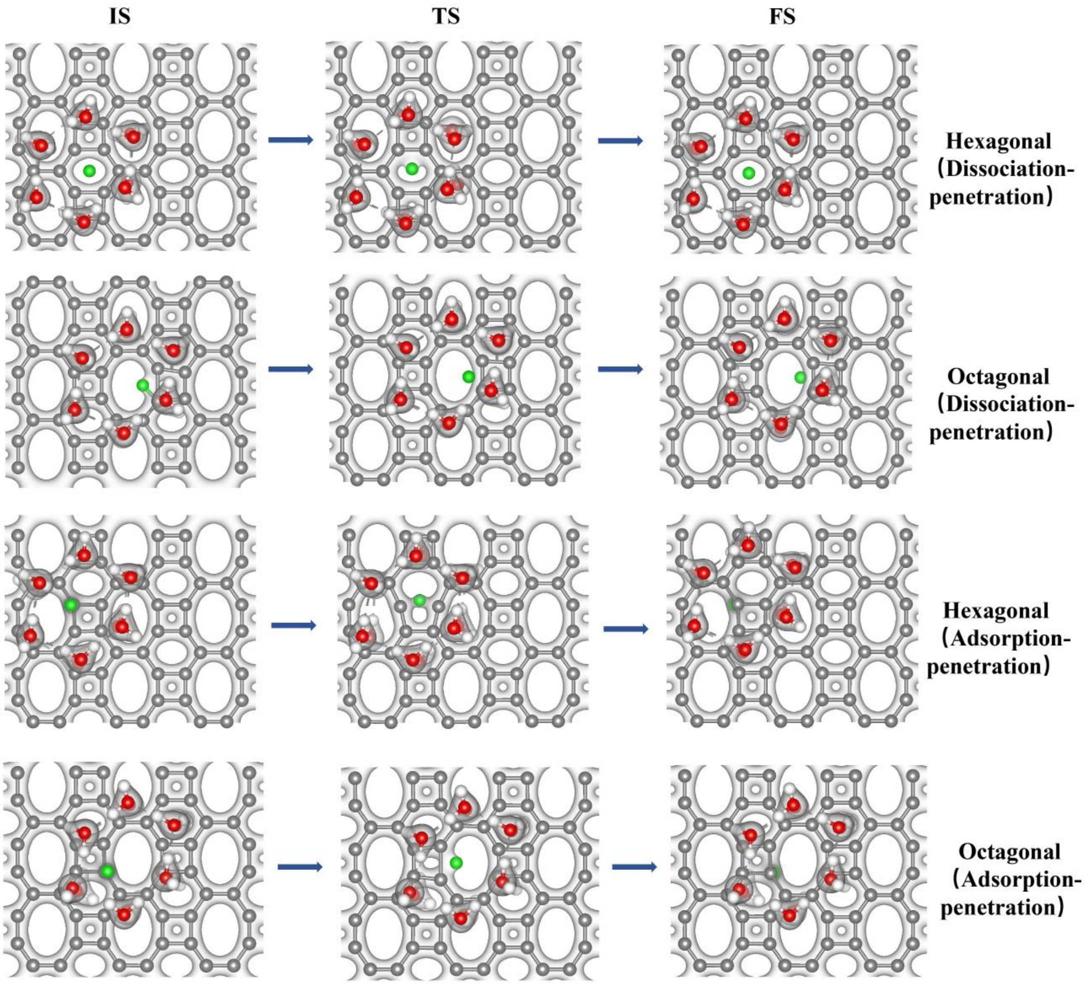

Fig. S7 Electron density clouds at an isosurface of 0.15 $e^-/Bohr^3$ for proton penetration through the biphenylene layer in aqueous environment.

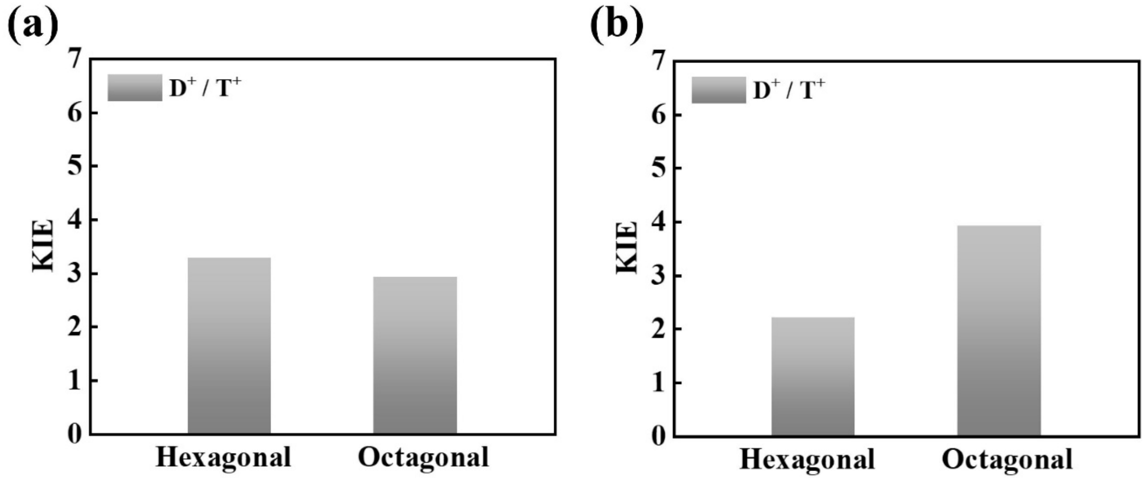

Fig. S8 Kinetic isotope effects of D^+/T^+ for the proton transmission within path 1 mode in vacuum environment (a) and dissociation-penetration pattern in aqueous environment (b).

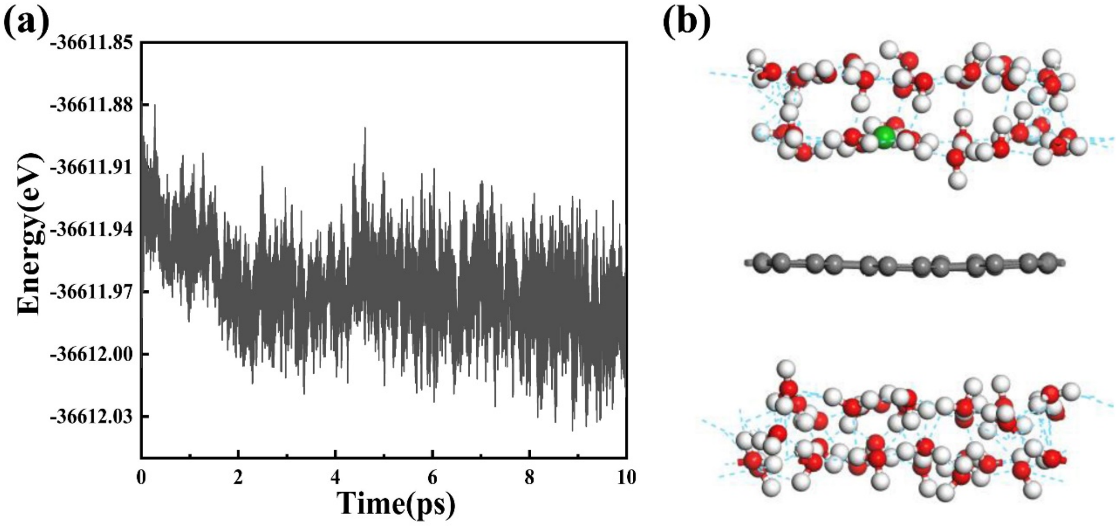

Fig. S9 (a) Ab initio molecular dynamics (AIMD) simulations in the NVT ensemble for the systems of the 2D biphenylene layer with larger water clusters. (b) Equilibrated structure with the lowest energy extracted from AIMD simulations.

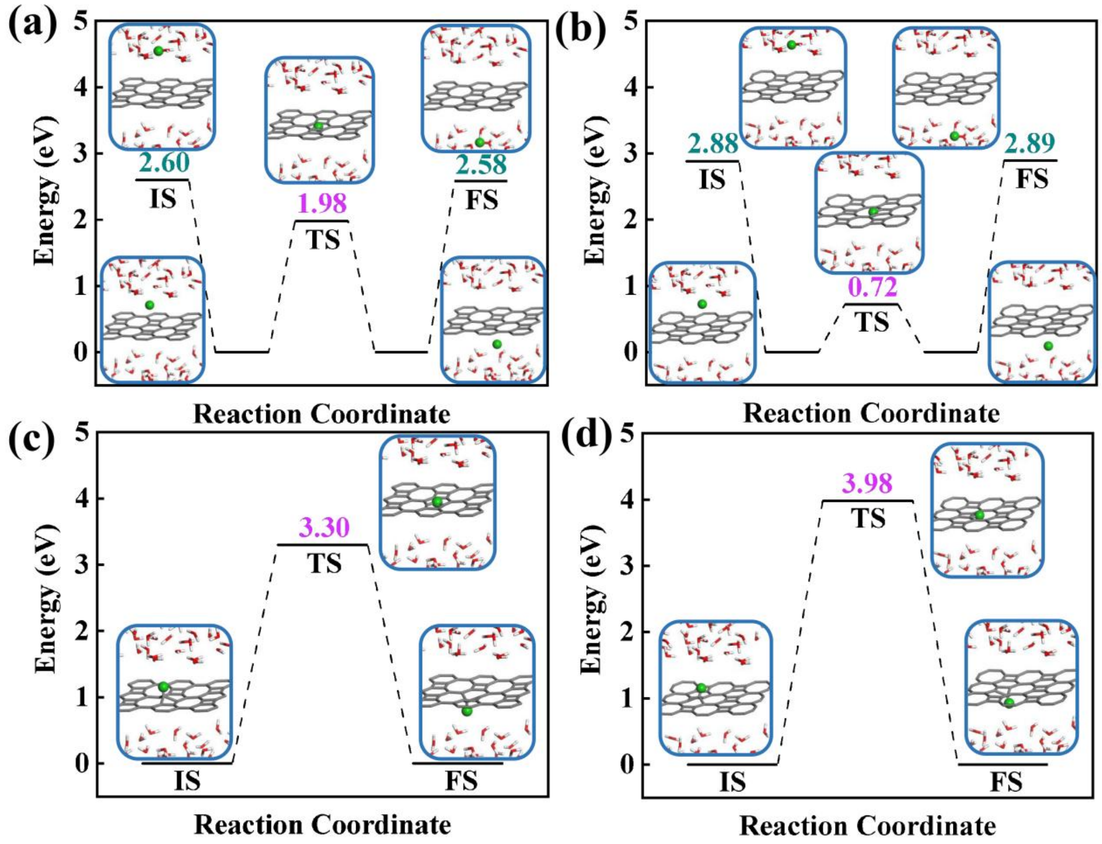

Fig. S10 Energy profiles of proton passing across biphenylene layer with larger water clusters. Proton penetration through the hexagonal ring (a) and octagonal ring (b) in the dissociation-penetration mode. Proton transfer through the hexagonal ring (c) and octagonal ring (d) in adsorption-penetration routine.

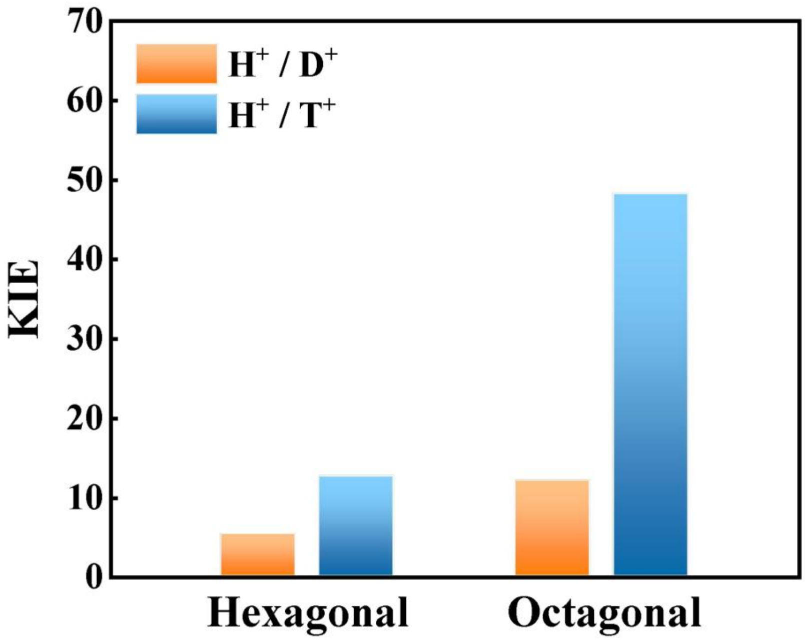

Fig. S11 Kinetic isotope effects of H^+/D^+ and H^+/T^+ for the proton transmission in dissociation-penetration pattern in aqueous environment with larger water clusters.

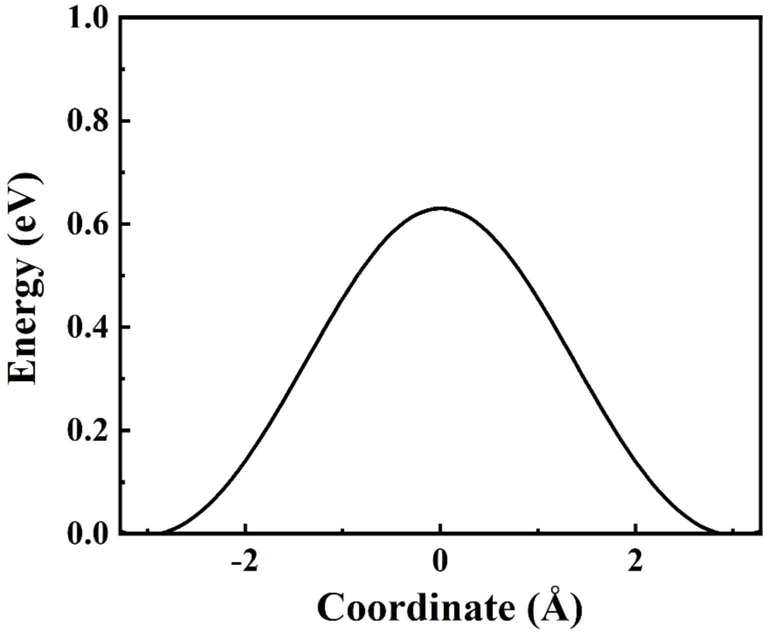

Fig. S12 Minimum energy path for the proton penetration through the octagonal ring in dissociation-penetration mode.

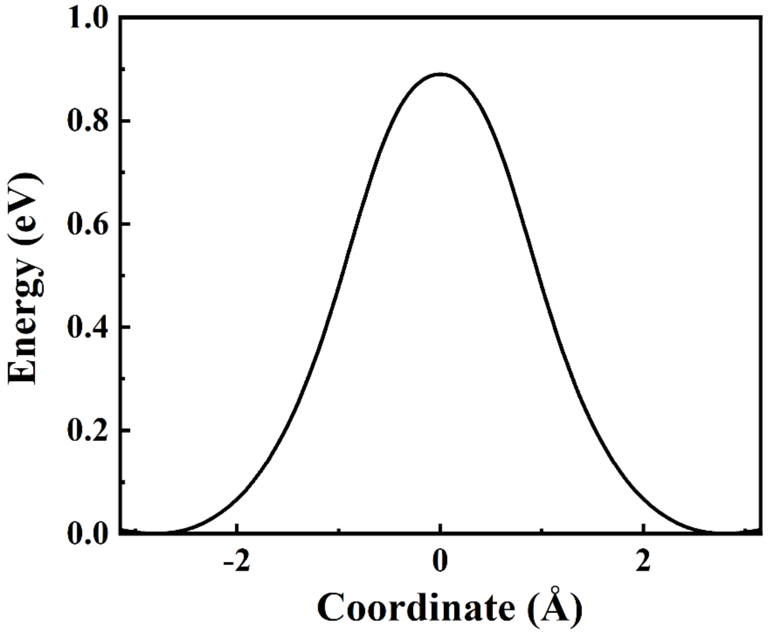

Fig. S13 Minimum energy path for the proton penetration through the hexagonal ring along path 1 mode.

Table S1. Kinetic isotope effects of H^+/D^+, H^+/T^+ and D^+/T^+ for the proton transmission in path 1 mode.

System	Hexagonal	Octagonal
H^+ / D^+	5.87	10.37
H^+ / T^+	19.17	30.33
D^+ / T^+	3.27	2.92

Table S2. Kinetic isotope effects of H^+/D^+, H^+/T^+ and D^+/T^+ for the proton transmission in dissociation-penetration pattern.

System	Hexagonal	Octagonal
H^+ / D^+	6.08	13.58
H^+ / T^+	13.36	53.10
D^+ / T^+	2.20	3.91

Table S3. The classical particle flow (u_{class}) and tunneling-contributed flow (u_{tunnel}) for H^+, D^+, and T^+ transmission across the octagonal ring in dissociation-penetration mode.

		Octagonal (Dissociation-penetration)
H^+	u_{class}	7.69×10^{-15}
	u_{tunnel}	9.96×10^{-32}
D^+	u_{class}	5.44×10^{-15}
	u_{tunnel}	1.82×10^{-44}
T^+	u_{class}	4.44×10^{-15}
	u_{tunnel}	1.57×10^{-50}
$u_{H+ / D+}$		1.41
$u_{H+ / T+}$		1.73

Table S4. The classical particle flow (u_{class}) and tunneling-contributed flow (u_{tunnel}) for H^+, D^+, and T^+ transmission across the hexagonal ring in path 1 mode.

		Hexagonal (Path 1)
H^+	u_{class}	3.33×10^{-19}
	u_{tunnel}	1.19×10^{-28}
D^+	u_{class}	2.36×10^{-19}
	u_{tunnel}	2.51×10^{-40}
T^+	u_{class}	1.92×10^{-19}
	u_{tunnel}	1.57×10^{-48}
$u_{H+ / D+}$		1.41
$u_{H+ / T+}$		1.73

The Fortran code to calculated *T(E)* and quantum tunneling contribution

```
! Program for calculating the flow rate

implicit none

! Variables

real , allocatable :: x(:),y(:)          ! z direction and potentials
real                :: interval          ! step interval for trapezoidal integration
real                :: integral          ! integral dummy variable for calculating T(E)
real                :: t_cofficient      ! Transmission coefficient
real                :: temp              ! temperature for calculating beta
real                :: beta              ! beta = 1/k_b*T k_b is Boltzmann constant
real                :: integral_2        ! dummy integral variable for calculating the particle
flow rate

real                :: u_tunnel, u_class, u_total     ! particle flow rate for tunneling, classic,
and total.

! Dummy variables

real :: i, j
integer:: m, n

! Constants

real                :: k
real                :: pi
real                :: mass
```

```
k        = 3.166E-06            ! Boltzmann constant
pi       = 3.14                 ! pi

! Reading the mass

write(*,*)    "what is the mass of the particle in atomic units (au)"

read(*,*)    mass

mass = mass*1837

!########################################################## Reading the potential

open(unit=1, file="potential.txt")
open(unit=2, file="transmission.txt")
! Reading the allocation size

i=0

do
read(1,*, end = 5) j
i=i+1
end do

! Allocating the size
5 continue
allocate(x(int(i)),y(int(i)))
close(1)
```

```
! Reading the potential

open(unit=1, file="potential.txt")


do m=1,int(i)

read(1,*) x(m),y(m)

!write(*,*) x(m), y(m)

end do


!######################################################## Calculating the transmission
coefficient


! Calculating the integral first


interval=5.7/int(i)                ! 6.4 for 0.63 eV U(z) dataset,    5.70 for 0.89 eV U(z) dataset


integral=0


do m=1,int(i)


if (m==1 .or. m==int(i)) then

integral=integral+sqrt(2*mass*0.0367*(y(m)))


else

integral=integral+2*sqrt(2*mass*0.0367*(y(m)))

end if


end do
```

```
integral=(interval*integral)/2.0

t_cofficient=exp(-2*integral)

!#DEBUG:
!write(*,*) t_cofficient
!STATUS: WORKING

!######################################################## Calculating the second integral

! Calculating beta

temp=300.0

beta= 1/(k*temp)

integral_2 =0.0

do m = 1, int(i)
integral_2 = integral_2+ t_cofficient* exp(-beta*y(m)) * (y(m+1)-y(m))
end do

u_tunnel = sqrt(beta/(2*pi))*integral_2
u_class    = exp(-beta*0.89*0.0367)/sqrt(2*pi*mass*beta)      ! 0.63 is the U_max which changes for 0.89 for data set 2
u_total    = u_class+u_tunnel

write(*,*) u_total, u_class, u_tunnel
```

end

Coordinates of initial state (IS) and transition state (TS) for proton penetration
Hexagonal (Path1)
IS

C	0.7424692987	1.7309170883	7.4996704122
C	3.7922614795	3.1630499737	7.4999776004
C	3.7924533660	1.7311253344	7.4998578925
C	0.7425454505	3.1631859007	7.4996657122
C	1.5509584994	0.5756223532	7.4996852647
C	2.9841337773	0.5756042380	7.4997188822
C	5.2777470467	1.7311258861	7.4998576738
C	8.3276580374	3.1631880094	7.4996660639
C	8.3277342031	1.7309144078	7.4996710208
C	5.2779388783	3.1630493201	7.4999768696
C	6.0860666494	0.5756041525	7.4997195234
C	7.5192415887	0.5756221076	7.4996851939
C	9.8126677029	1.7309640751	7.4996943370
C	12.8627345741	3.1632096674	7.4996353778
C	12.8628267359	1.7309673155	7.4996934634
C	9.8127599085	3.1632124265	7.4996365594
C	10.6211040570	0.5756840169	7.4996815550
C	12.0543937826	0.5756841850	7.4996823168
C	0.7425516032	5.4736905106	7.4997207253
C	3.7923889327	6.9056702702	7.4998015474
C	3.7922545832	5.4738311041	7.5000337890
C	0.7425344120	6.9058737331	7.4996134755
C	1.5508763206	4.3183996193	7.4997577741
C	2.9841014890	4.3184013335	7.5000887825
C	5.2779458305	5.4738316609	7.5000335691
C	8.3276690737	6.9058758202	7.4996138597
C	8.3276518800	5.4736878315	7.4997213031
C	5.2778114322	6.9056696298	7.4998008155
C	6.0860989142	4.3184012472	7.5000894040
C	7.5193237654	4.3183993649	7.4997577167
C	9.8127665674	5.4736633401	7.4996913709
C	12.8627620579	6.9058235259	7.4996368097
C	12.8627278603	5.4736665709	7.4996904857
C	9.8127324464	6.9058262634	7.4996379820
C	10.6211008608	4.3183990888	7.4996889055
C	12.0543969655	4.3183992542	7.4996896803
C	0.7423574274	9.2162283817	7.4996902525
C	3.7926156365	10.6486697235	7.4996540795
C	3.7926111162	9.2162345056	7.4997095570
C	0.7423532822	10.6486723644	7.4996346877

C	1.5509575699	8.0612156957	7.4996845966
C	2.9841344041	8.0612369428	7.4997180032
C	5.2775892985	9.2162350711	7.4997093320
C	8.3278501971	10.6486744592	7.4996350847
C	8.3278460492	9.2162256907	7.4996908751
C	5.2775847238	10.6486690764	7.4996533410
C	6.0860660145	8.0612368636	7.4997186451
C	7.5192425160	8.0612154351	7.4996845254
C	9.8126432663	9.2161857780	7.4996931579
C	12.8628557262	10.6487116207	7.4996367629
C	12.8628511806	9.2161890400	7.4996922609
C	9.8126387740	10.6487143694	7.4996379543
C	10.6211031470	8.0611531873	7.4996813426
C	12.0543947035	8.0611533570	7.4996821066
H	4.5350542870	4.3176734630	12.4956356296

TS

C	0.7583990035	1.7169261554	10.0018369363
C	3.7082413811	3.1559844733	10.0043750260
C	3.8199210097	1.7611329052	10.0033167637
C	0.7180397041	3.1564977267	10.0021574428
C	1.5838405025	0.5833454512	10.0022932450
C	3.0177362606	0.5812902657	10.0028345185
C	5.2993678947	1.7611145183	10.0016149129
C	8.4012415265	3.1565064568	9.9989898215
C	8.3608970012	1.7169189933	9.9988642794
C	5.4110471907	3.1559742107	10.0022601829
C	6.1015506244	0.5812782181	9.9998609194
C	7.5354519105	0.5833424156	9.9986208560
C	9.8491094802	1.7130383412	9.9973349729
C	12.8705270619	3.1538644147	9.9990267269
C	12.8755248778	1.7130212764	9.9988131352
C	9.8540541716	3.1538743901	9.9974683600
C	10.6467454851	0.5686355497	9.9955171773
C	12.0778693631	0.5686179185	9.9961103072
C	0.7180087489	5.4825777613	10.0002914036
C	3.8199077349	6.8777701944	10.0003906146
C	3.7085326908	5.4828587132	10.0024498109
C	0.7583946022	6.9219756361	9.9987190878
C	1.4975595709	4.3195279198	10.0032539475
C	2.9053423454	4.3195311691	10.0043033521
C	5.4107441895	5.4828589618	10.0003407928
C	8.3609041071	6.9220110488	9.9957484576
C	8.4012730717	5.4826095839	9.9971163421

C	5.2993857852	6.8777839299	9.9987078344
C	6.2139380043	4.3195357412	10.0006707767
C	7.6217209092	4.3195445047	9.9990882010
C	9.8541266669	5.4851606134	9.9955936220
C	12.8753906970	6.9258619315	9.9956932827
C	12.8704561029	5.4851454315	9.9971651209
C	9.8492317049	6.9258752172	9.9942108678
C	10.6524961358	4.3194692381	9.9960340737
C	12.0720790602	4.3194560680	9.9966662917
C	0.7814623421	9.2261010876	9.9981320483
C	3.8177845527	10.6571969440	10.0008672409
C	3.8177940083	9.2098047859	9.9997070284
C	0.7814247001	10.6409793965	9.9993906830
C	1.5838211922	8.0556363289	9.9997510240
C	3.0177334507	8.0576343901	10.0003831817
C	5.3015051996	9.2098124070	9.9982679604
C	8.3378862014	10.6409913746	9.9964463232
C	8.3378478701	9.2261139912	9.9951946574
C	5.3015090922	10.6571808223	9.9994053835
C	6.1015578512	8.0576345152	9.9974356202
C	7.5354721955	8.0556519394	9.9961243929
C	9.8351892312	9.2273118023	9.9935276267
C	12.8894545148	10.6398039778	9.9963524053
C	12.8894290159	9.2272930200	9.9950970208
C	9.8351559245	10.6398255255	9.9947880776
C	10.6467365054	8.0704115484	9.9929572589
C	12.0778909817	8.0704027933	9.9935656572
H	4.5597419404	4.3275336773	10.0042867266

Octagonal (Path1)

IS

C	0.7395173274	1.7276656660	9.8771037043
C	3.7957441545	3.1665997524	9.8820009930
C	3.7956931969	1.7274713874	9.8804327309
C	0.7394743697	3.1663704254	9.8778799406
C	1.5492972261	0.5770193203	9.8769067585
C	2.9857612288	0.5770339616	9.8783133937
C	5.2745991286	1.7274412750	9.8834890364
C	8.3303964668	3.1665680331	9.8881801487
C	8.3303422781	1.7275956972	9.8852317867
C	5.2745762504	3.1666837800	9.8855896574
C	6.0843054290	0.5770573800	9.8839257058
C	7.5207135683	0.5770283475	9.8845844041

C	9.8093871516	1.7275543533	9.8831042845
C	12.8652515477	3.1664347604	9.8789531892
C	12.8652462818	1.7276082554	9.8780475626
C	9.8093388953	3.1665470418	9.8856128543
C	10.6191310794	0.5769585834	9.8807129301
C	12.0555811518	0.5769585743	9.8786506030
C	0.7421369563	5.4747542645	9.8788923473
C	3.7921088274	6.9061616537	9.8831214355
C	3.7930104628	5.4747158173	9.8837341993
C	0.7428664098	6.9060768255	9.8786083235
C	1.5493398351	4.3169333320	9.8787901833
C	2.9857312861	4.3169366925	9.8810199892
C	5.2772906444	5.4747027502	9.8879323402
C	8.3270140538	6.9061890518	9.8906824917
C	8.3277387852	5.4745942126	9.8919859096
C	5.2781385944	6.9061840258	9.8870841708
C	6.0844160598	4.3169439375	9.8893564733
C	7.5206879853	4.3169926323	9.8911858578
C	9.8118691311	5.4746610000	9.8886460340
C	12.8619558079	6.9061051874	9.8798144954
C	12.8626883278	5.4748086157	9.8801193986
C	9.8126593060	6.9062026765	9.8876749691
C	10.6191179756	4.3170446876	9.8850824909
C	12.0554761884	4.3170096978	9.8812594129
C	0.7428951556	9.2158737498	9.8771970541
C	3.7928767697	10.6472081571	9.8796318386
C	3.7920640753	9.2158139138	9.8806193091
C	0.7422439821	10.6471876626	9.8767086776
C	1.5496476478	8.0609759471	9.8780836398
C	2.9852335982	8.0609692970	9.8799964767
C	5.2781806009	9.2157389360	9.8837830720
C	8.3276059534	10.6473171421	9.8838812088
C	8.3269613844	9.2157088206	9.8857399760
C	5.2773751811	10.6472215585	9.8824913050
C	6.0848440583	8.0609506465	9.8872647650
C	7.5203665199	8.0609279791	9.8886681972
C	9.8127683560	9.2157354813	9.8835752008
C	12.8626183958	10.6471396598	9.8776045106
C	12.8619074501	9.2158224436	9.8781980499
C	9.8120660633	10.6472423561	9.8819282848
C	10.6195835986	8.0609203281	9.8836345345
C	12.0550952549	8.0609543144	9.8804089363
H	7.3694005721	6.2011430000	11.9384187629

TS

C	0.7409100332	1.7287538368	9.8726879231
C	3.7946010365	3.1644722471	9.8715820304
C	3.7946033931	1.7285256943	9.8719264272
C	0.7409203898	3.1642344448	9.8725440787
C	1.5468400025	0.5752264056	9.8727605266
C	2.9883140894	0.5752382677	9.8724397807
C	5.2757702358	1.7285753077	9.8712315267
C	8.3294351471	3.1644332316	9.8701569240
C	8.3293638847	1.7286819310	9.8708327380
C	5.2757323277	3.1645526944	9.8707647055
C	6.0818364251	0.5752327767	9.8711996866
C	7.5234297526	0.5752225495	9.8710575487
C	9.8108597927	1.7286462133	9.8713175089
C	12.8643672569	3.1642787791	9.8722969374
C	12.8643721012	1.7287658816	9.8724745055
C	9.8108285034	3.1643967791	9.8707375257
C	10.6170120493	0.5752023571	9.8719128283
C	12.0584510856	0.5752029149	9.8723764610
C	0.7409866818	5.4714156134	9.8723006701
C	3.7944443989	6.9070203629	9.8713837198
C	3.7945391810	5.4711701826	9.8711832798
C	0.7410567378	6.9067905726	9.8723869944
C	1.5468613310	4.3177533993	9.8723475414
C	2.9882899064	4.3177500943	9.8718315907
C	5.2757366114	5.4712060503	9.8702731407
C	8.3293848696	6.9069697585	9.8697388106
C	8.3294598700	5.4712364090	9.8692709433
C	5.2758130634	6.9070036454	9.8705307159
C	6.0819020402	4.3178194060	9.8699362345
C	7.5233921284	4.3178414818	9.8695081856
C	9.8107871493	5.4712540397	9.8700430702
C	12.8643205641	6.9067954006	9.8721117597
C	12.8643690434	5.4714657455	9.8720156860
C	9.8108411649	6.9070058879	9.8703583854
C	10.6170333743	4.3178434785	9.8708734117
C	12.0584035262	4.3178171776	9.8717661572
C	0.7410370458	9.2142534783	9.8726718654
C	3.7945028668	10.6498344806	9.8721562431
C	3.7944559333	9.2140510322	9.8719276615
C	0.7410007143	10.6496416469	9.8728086892
C	1.5468061264	8.0605504872	9.8725020520
C	2.9883292162	8.0605378330	9.8720731736

C	5.2758695862	9.2139672135	9.8712761387
C	8.3292718198	10.6496992514	9.8712735507
C	8.3293180741	9.2140468488	9.8708610458
C	5.2758714983	10.6497789936	9.8715682453
C	6.0818547565	8.0604740394	9.8705054870
C	7.5234427595	8.0604576370	9.8702072143
C	9.8109060784	9.2140788522	9.8713014716
C	12.8643042097	10.6495863623	9.8726186611
C	12.8642936244	9.2141961331	9.8724585450
C	9.8109286146	10.6497052429	9.8716714868
C	10.6169996102	8.0604813358	9.8712712549
C	12.0584416109	8.0605105812	9.8719866431
H	7.3775251580	6.2011430000	9.9722982333

Hexagonal (Path2)
IS

C	0.7548915787	1.7385301120	9.3346890465
C	3.8349000959	3.1889059254	9.2664212352
C	3.8349883240	1.7224307108	9.2185224246
C	0.7569740809	3.1722350559	9.3830771170
C	1.5627633532	0.5820490411	9.3042133397
C	3.0034923620	0.5748570734	9.2591720928
C	5.2723911531	1.7107815256	9.2253147418
C	8.3290320262	3.1697300015	9.3649948713
C	8.3297015661	1.7361000492	9.3488892010
C	5.2780596198	3.2016911836	9.2632420343
C	6.0811116834	0.5752129343	9.2981127250
C	7.5208796382	0.5780522758	9.3461988146
C	9.8141659666	1.7359313974	9.3680382676
C	12.8734021351	3.1725154009	9.3945515348
C	12.8717218451	1.7391333183	9.3572583570
C	9.8161015917	3.1694372473	9.3847701561
C	10.6255439126	0.5816302177	9.3721503197
C	12.0563832041	0.5862098731	9.3601330100
C	0.7569815168	5.4856290689	9.4807194292
C	3.7646472109	6.9124287061	10.0929798864
C	3.8301330054	5.4577340686	9.6099032861
C	0.7531853686	6.9152157111	9.4868687765
C	1.5668818683	4.3279312671	9.4342622543
C	3.0104959140	4.3285437016	9.4173138762
C	5.2500365933	5.4971310804	9.5343312557
C	8.3243893133	6.9057423175	9.4766532968
C	8.3265693713	5.4808642184	9.4482769554

C	5.2723043738	6.9026691151	9.7584147659
C	6.0839724813	4.3249784275	9.3653453715
C	7.5179273908	4.3231110028	9.3902891848
C	9.8197650077	5.4810480754	9.4410545485
C	12.8650419933	6.9101201735	9.4554085229
C	12.8704692874	5.4837052815	9.4604085159
C	9.8181650954	6.9079162036	9.4511210559
C	10.6289925844	4.3227516824	9.4178374865
C	12.0610641387	4.3236613265	9.4272777289
C	0.7425408675	9.2265316215	9.3806919076
C	3.8148329775	10.6528814938	9.3071516319
C	3.7867049050	9.1853949763	9.4603357669
C	0.7480844861	10.6546393347	9.3369177494
C	1.5540992785	8.0754903534	9.4754365671
C	2.9809004143	8.0903531439	9.5725358816
C	5.2573321407	9.2027268409	9.5261847272
C	8.3275826109	10.6512959004	9.3882569911
C	8.3222486862	9.2187434339	9.4298215699
C	5.2579015996	10.6404894425	9.3492868913
C	6.0774950171	8.0591994405	9.6181493045
C	7.5157124772	8.0639014870	9.4984192670
C	9.8130632871	9.2200783075	9.4104933070
C	12.8652074514	10.6567815253	9.3518149683
C	12.8609208887	9.2249977461	9.3801999254
C	9.8155177982	10.6499809458	9.3846306590
C	10.6241651623	8.0649399607	9.4258942079
C	12.0547690022	8.0669898147	9.4189698606
H	3.6190529160	6.8936549512	11.1888968886

TS

C	0.7511698085	1.7500013630	9.9954997813
C	3.7902387854	3.1576295811	9.9947198231
C	3.8067924045	1.7005034906	9.9938480338
C	0.7388699068	3.1803510950	9.9960341746
C	1.5514750164	0.5875790439	9.9950426207
C	2.9899349571	0.5563895259	9.9944408908
C	5.2605347909	1.7000066043	9.9938700291
C	8.3164728416	3.1858272825	9.9955578598
C	8.3190611239	1.7570598713	9.9955668609
C	5.2676325563	3.1584078886	9.9944971913
C	6.0773778796	0.5548013847	9.9947948017
C	7.5166013425	0.5849870557	9.9954725360
C	9.7970190121	1.7643100669	9.9959616802
C	12.8703902927	3.1818561377	9.9962142517

C	12.8648538718	1.7559614553	9.9958639283
C	9.8020788024	3.1880923675	9.9959566059
C	10.6188567691	0.6165596064	9.9960339796
C	12.0460486833	0.6174230970	9.9958752089
C	0.7364749565	5.4868757533	9.9968878124
C	3.4927027087	6.8212284859	10.0051089986
C	3.7865707200	5.4801514339	9.9994849432
C	0.6966456443	6.9360520359	9.9969463506
C	1.5536830020	4.3343757180	9.9966575385
C	2.9859704218	4.2910755099	9.9968122158
C	5.2686814007	5.4809405666	9.9983623908
C	8.3647664884	6.9297309623	9.9966213834
C	8.3149323580	5.4886616664	9.9961840552
C	5.5652432460	6.8227110297	10.0024398489
C	6.0682294643	4.2921923495	9.9958204707
C	7.5024214841	4.3360566652	9.9957703528
C	9.8113292131	5.4835825074	9.9960654428
C	12.8445768232	6.9251079315	9.9961979431
C	12.8612910726	5.4819844623	9.9964628343
C	9.8207043885	6.9154697831	9.9960690938
C	10.6196246372	4.3370868815	9.9962137490
C	12.0546352369	4.3363662621	9.9964207602
C	0.6797448834	9.2601455396	9.9957731346
C	3.7984414112	10.6182667554	9.9954190170
C	3.7665578716	9.1788840268	9.9982359553
C	0.7173701994	10.6842694675	9.9952701991
C	1.4638680978	8.1059899391	9.9976623612
C	2.8761799381	8.0929382262	10.0007850438
C	5.2938380705	9.1795075264	9.9984193896
C	8.3588960192	10.6925461477	9.9958349049
C	8.3919869717	9.2511342629	9.9961803861
C	5.2667840714	10.6169215824	9.9956922406
C	6.1852773626	8.0954083243	10.0003084401
C	7.5986217441	8.1100357357	9.9976992453
C	9.8361295133	9.2428502642	9.9957429233
C	12.8344164384	10.6882961323	9.9953833708
C	12.8286147927	9.2558211438	9.9954724249
C	9.8268832536	10.6986358215	9.9957957674
C	10.6247507030	8.0794332556	9.9958990395
C	12.0435576079	8.0789149662	9.9958500771
H	4.5300994027	7.3916781812	9.9936210434

Octagonal (Path2)

IS

C	0.7429754474	1.7478953427	9.4142546244
C	3.8126094889	3.1972371299	9.5800267115
C	3.8125933974	1.7600265960	9.4016031998
C	0.7478328911	3.1810911961	9.4572958705
C	1.5499791108	0.5938596312	9.3783480014
C	2.9898168247	0.5975651876	9.3425277739
C	5.2555837394	1.7474623655	9.3611676800
C	8.3280877493	3.1736350903	9.4150000442
C	8.3221950866	1.7447701435	9.3666131160
C	5.2839039421	3.2143420102	9.5168220788
C	6.0668363733	0.5978964169	9.3053737756
C	7.5077585270	0.5902443041	9.3387466951
C	9.8103159738	1.7428147558	9.3687285394
C	12.8632958624	3.1797093800	9.4273139648
C	12.8604376556	1.7493853296	9.3991147893
C	9.8136621134	3.1751129847	9.4008795357
C	10.6194178726	0.5859954657	9.3685177842
C	12.0504873270	0.5903234290	9.3798860692
C	0.7453662574	5.4938601378	9.5030330507
C	3.8205031420	6.9033474542	9.5791804228
C	3.7973465306	5.4976847129	9.8050171932
C	0.7440736379	6.9195044813	9.4717597047
C	1.5542028597	4.3359492878	9.5331981194
C	2.9916136961	4.3411603948	9.6639880508
C	5.3055451636	5.4886995186	10.1415874330
C	8.3139128949	6.9150814548	9.5115787825
C	8.3182378837	5.4847864163	9.5220177294
C	5.2401008739	6.9427666416	9.6563776606
C	6.0900755382	4.3096207902	9.6244341411
C	7.5166026695	4.3244076098	9.5184842209
C	9.8101972559	5.4896871555	9.4771375944
C	12.8563149790	6.9190243658	9.4548996350
C	12.8582178561	5.4917880838	9.4668447947
C	9.8054272042	6.9168149508	9.4785759414
C	10.6208518923	4.3330331559	9.4358127516
C	12.0513342794	4.3348682106	9.4401116455
C	0.7423446480	9.2307733889	9.3858398700
C	3.7986223882	10.6900394173	9.2708268295
C	3.7925528769	9.1987961377	9.3070910652
C	0.7416868885	10.6628481620	9.3705902940
C	1.5530949017	8.0765160312	9.4203003000

C	2.9870160615	8.0752814957	9.4062358671
C	5.2356440484	9.2113472478	9.3108792840
C	8.3150735834	10.6606281716	9.3581557859
C	8.3131383880	9.2282888144	9.4061319989
C	5.2355095068	10.6779978829	9.2649020737
C	6.0597943032	8.0711283715	9.4603014745
C	7.5036510675	8.0716811816	9.4679887746
C	9.8029619966	9.2279645697	9.4059847854
C	12.8611030671	10.6631336372	9.3779879785
C	12.8592295955	9.2307560604	9.3940754041
C	9.8048087374	10.6600710703	9.3679473415
C	10.6149321136	8.0755337548	9.4368268379
C	12.0470063070	8.0764961541	9.4255080066
H	5.4495265411	5.5110003266	11.2375907561

TS

C	0.7362280986	1.7328515405	10.0000968021
C	3.8139495839	3.1751769617	10.0000729087
C	3.7985142657	1.6984067086	10.0001408771
C	0.7496572080	3.1722348063	10.0000905527
C	1.5391913229	0.5786272606	10.0001077694
C	2.9714205813	0.5785765841	10.0001338738
C	5.2231057477	1.6808808738	10.0001419928
C	8.3546112610	3.1585688373	10.0000901525
C	8.3484989514	1.7254600167	10.0001096369
C	5.2432184811	3.1266185092	10.0000635710
C	6.0785289351	0.5785837458	10.0001414039
C	7.5222785513	0.5786218135	10.0001201967
C	9.8235040341	1.7281047766	10.0001104220
C	12.8742588432	3.1741317104	10.0000966453
C	12.8706179714	1.7352976038	10.0000975593
C	9.8321048458	3.1671304221	10.0001085152
C	10.6368042857	0.5786218012	10.0001031262
C	12.0615980532	0.5786520514	10.0000980114
C	0.7566908826	5.4758841945	10.0000759158
C	3.8588909262	6.9271913369	10.0000779381
C	3.8589304496	5.4581197108	9.9999300859
C	0.7566069363	6.9095788041	10.0000826383
C	1.5690643815	4.3210231518	10.0000661755
C	3.0066732304	4.3122271247	10.0000226292
C	5.2197994182	5.2630554152	9.9997729383
C	8.3140073360	6.9094955332	10.0000546124
C	8.3139769652	5.4759076670	10.0000475337
C	5.2197598826	7.1223040155	10.0000421916

C	6.1075461020	4.2038552041	9.9999368516
C	7.5356900836	4.2879086841	10.0000205937
C	9.8104447203	5.4817866255	10.0000871670
C	12.8797963500	6.9092265400	10.0000886278
C	12.8798895485	5.4761845511	10.0000874248
C	9.8104416842	6.9036347242	10.0000860664
C	10.6286471199	4.3222881965	10.0000996229
C	12.0631531511	4.3247730128	10.0000957488
C	0.7495976976	9.2131987532	10.0001009048
C	3.7984731214	10.6868578093	10.0001482574
C	3.8139315523	9.2100860624	10.0001183460
C	0.7361808911	10.6525403611	10.0001034365
C	1.5689803538	8.0644189840	10.0000968581
C	3.0066060237	8.0730934369	10.0001061949
C	5.2432063584	9.2587135994	10.0000937519
C	8.3485300173	10.6599496328	10.0001070448
C	8.3545400466	9.2268400836	10.0000851816
C	5.2230551273	10.7044172595	10.0001442623
C	6.1075596253	8.1814903019	10.0000249618
C	7.5356903369	8.0974718417	10.0000525165
C	9.8320174805	9.2182575580	10.0000930790
C	12.8705831611	10.6501053014	10.0000979920
C	12.8741869472	9.2112877474	10.0000971921
C	9.8235137863	10.6572616332	10.0001044103
C	10.6285909526	8.0631682762	10.0000933502
C	12.0630690399	8.0606619946	10.0000960052
H	6.0022504557	6.1926753837	10.0002064030

Hexagonal (Dissociation-penetration)

IS

C	0.7921827099	1.7286477224	9.8727583318
C	3.8459518994	3.1644861504	9.8728649040
C	3.8459766305	1.7284522227	9.8728157877
C	0.7922558616	3.1643393193	9.8727777031
C	1.5982016972	0.5752584459	9.8728369616
C	3.0396963403	0.5752447081	9.8727909940
C	5.3269976793	1.7285350221	9.8727856799
C	8.3806988763	3.1643005929	9.8728765061
C	8.3806868317	1.7287756815	9.8728154550
C	5.3269750979	3.1645806490	9.8727917572
C	6.1332678207	0.5753010405	9.8728617637
C	7.5747304957	0.5752517025	9.8728010279
C	9.8624149789	1.7287095893	9.8728033812

C	12.9159212968	3.1643178183	9.8728736604
C	12.9159750337	1.7286173534	9.8728110519
C	9.8624612874	3.1642617852	9.8728176234
C	10.6684547665	0.5752261594	9.8728469316
C	12.1097830033	0.5751822174	9.8727844464
C	0.7923781435	5.4714911481	9.8727895660
C	3.8457642579	6.9070519769	9.8728689187
C	3.8457954107	5.4712931205	9.8728047687
C	0.7924153337	6.9068815289	9.8727888121
C	1.5981982472	4.3177628222	9.8728478135
C	3.0397119791	4.3177270759	9.8728135062
C	5.3270466381	5.4712894907	9.8727756605
C	8.3805547690	6.9067636016	9.8728847079
C	8.3806040306	5.4714956005	9.8728394667
C	5.3271592257	6.9070099014	9.8728043878
C	6.1332616886	4.3178052665	9.8728924052
C	7.5746966581	4.3177733470	9.8728292860
C	9.8625507936	5.4715595204	9.8727940314
C	12.9157912614	6.9068770743	9.8728661423
C	12.9157894698	5.4715189922	9.8728124012
C	9.8625749487	6.9068664615	9.8728113114
C	10.6684346048	4.3177969367	9.8728648467
C	12.1097914899	4.3177500590	9.8727944848
C	0.7923354927	9.2141645663	9.8727709771
C	3.8458849873	10.6498749783	9.8728570314
C	3.8458017648	9.2140597485	9.8728068520
C	0.7922587122	10.6498102241	9.8727957188
C	1.5981719626	8.0606699696	9.8728362186
C	3.0397321621	8.0606121025	9.8727596196
C	5.3271650691	9.2140239634	9.8727825576
C	8.3806045640	10.6496587617	9.8728887817
C	8.3805478693	9.2142213610	9.8728203565
C	5.3271195198	10.6498809888	9.8727916558
C	6.1332437838	8.0605808492	9.8728220295
C	7.5747505138	8.0605601101	9.8727894129
C	9.8625011233	9.2142753708	9.8727830253
C	12.9159568252	10.6497427892	9.8728608659
C	12.9158837862	9.2141419873	9.8728036598
C	9.8624633138	10.6496598590	9.8727952729
C	10.6684220608	8.0606667188	9.8728500626
C	12.1098009969	8.0606525504	9.8727904383
H	1.1369387599	2.3477694544	17.7006942333
H	1.7191925558	3.7783873649	17.6142122241
O	1.6284951439	2.9429781424	17.1127552023

H	4.9554024483	2.2346041855	17.0464601400
H	3.4159836449	2.0486407293	17.0725191616
O	4.2550071781	1.5468801316	17.1065184694
H	1.2114347835	6.2158423212	17.3778576308
H	2.3287995545	5.5550744790	16.5584103603
O	2.0624850874	5.7550935074	17.4748720687
H	3.6651966280	6.9777564879	17.4382791218
H	4.4425882586	8.2796370506	17.1053838901
O	4.3983356617	7.3323690837	16.8926813803
H	7.4981987825	6.5347594771	16.8934652841
H	6.0006319375	6.5481209693	17.2976479243
O	6.8616748468	6.0964623839	17.4783327131
H	6.6316038953	4.3222552806	17.1254026993
H	7.2119978758	2.9092972653	17.3842701359
O	6.5197492581	3.3679017673	16.8839112809
H	0.9767531316	2.4117929199	2.1247094833
H	1.5708718808	3.7947772251	2.4975917111
O	1.4795131845	2.8707681147	2.8152955334
H	4.6522862786	2.1846027312	2.8935005495
H	3.1309418387	1.9629075893	2.9985359230
O	3.9861240734	1.4822463087	3.0367431418
H	0.9147279903	6.1135664438	2.6259930903
H	2.1802235090	5.7323152911	3.4121729380
O	1.7820711295	5.6860425087	2.5234433148
H	3.5207547394	6.8939089730	2.1830949934
H	4.3385968600	8.1784989026	2.4463789092
O	4.2493524432	7.2516435221	2.7284375787
H	7.3437114290	6.5921431371	2.8619913140
H	5.8886911912	6.5440029207	2.3433420556
O	6.8002842900	6.2131690404	2.1533055971
H	6.5757244391	4.4151102864	2.6100619815
H	6.9922783672	2.9207973877	2.6301184383
O	6.3699292771	3.5443957711	3.0330903551
H	5.2419783721	3.7981085700	13.7185952471

TS

C	0.7921339303	1.7286125640	9.8727043302
C	3.8460145622	3.1645295030	9.8727832876
C	3.8460421327	1.7284055135	9.8727231611
C	0.7922088944	3.1643711938	9.8727125311
C	1.5982113349	0.5752623482	9.8728264589
C	3.0396886002	0.5752491431	9.8727570856
C	5.3269333137	1.7284883880	9.8727063777
C	8.3807512721	3.1643369553	9.8727940373

C	8.3807415278	1.7287353239	9.8727396695
C	5.3269145567	3.1646233276	9.8727207919
C	6.1332738886	0.5753064141	9.8728149344
C	7.5747219763	0.5752566048	9.8727647777
C	9.8623583663	1.7286702835	9.8726999351
C	12.9159689530	3.1643519512	9.8727963428
C	12.9160236206	1.7285832206	9.8727398900
C	9.8624054161	3.1643012846	9.8727162761
C	10.6684639342	0.5752291825	9.8728199738
C	12.1097730612	0.5751848173	9.8727702732
C	0.7923382120	5.4714605405	9.8727148991
C	3.8458148340	6.9070855874	9.8727648490
C	3.8458487631	5.4712570439	9.8727165870
C	0.7923770395	6.9069092328	9.8727221708
C	1.5982060043	4.3177571526	9.8728216834
C	3.0397053558	4.3177198184	9.8727415790
C	5.3269961889	5.4712521560	9.8726959133
C	8.3805961242	6.9067945832	9.8727994354
C	8.3806478282	5.4714599848	9.8727480393
C	5.3271121952	6.9070430595	9.8727123929
C	6.1332668990	4.3177970503	9.8728011137
C	7.5746900924	4.3177661543	9.8727605213
C	9.8624992964	5.4715275187	9.8726926300
C	12.9158337522	6.9069045142	9.8727884946
C	12.9158330086	5.4714907244	9.8727318955
C	9.8625250452	6.9068978344	9.8727049923
C	10.6684428625	4.3177939997	9.8728184030
C	12.1097820909	4.3177473241	9.8727706723
C	0.7922933760	9.2141384221	9.8727032091
C	3.8459457782	10.6499196415	9.8727787970
C	3.8458567689	9.2140246588	9.8727233320
C	0.7922132699	10.6498466438	9.8727308118
C	1.5981778968	8.0606740355	9.8728244746
C	3.0397263843	8.0606168426	9.8727282423
C	5.3271129310	9.2139899779	9.8726928941
C	8.3806549941	10.6497004948	9.8727987792
C	8.3805934427	9.2141920445	9.8727298646
C	5.3270603568	10.6499271004	9.8727055321
C	6.1332476225	8.0605859512	9.8727811592
C	7.5747460545	8.0605643686	9.8727555562
C	9.8624495953	9.2142424103	9.8726969041
C	12.9160031849	10.6497761144	9.8727939333
C	12.9159280609	9.2141131702	9.8727331882
C	9.8624095952	10.6496973085	9.8727118873

C	10.6684296556	8.0606689087	9.8728229968
C	12.1097925516	8.0606546004	9.8727753807
H	1.1047666773	2.3690629991	17.6942121252
H	1.7129341981	3.7887098108	17.5971853624
O	1.6293591270	2.9420512752	17.1129682232
H	4.9512228994	2.2340874633	17.0983196922
H	3.4107862213	2.0417867710	17.1119093542
O	4.2512343278	1.5452451450	17.1843718306
H	1.2096630201	6.2149821526	17.3772803412
H	2.3359520884	5.5665263222	16.5591163327
O	2.0609052490	5.7547261492	17.4755415201
H	3.6656579935	6.9752946650	17.4445760979
H	4.4603680924	8.2634662761	17.1050411540
O	4.4006819117	7.3163369704	16.8936170269
H	7.5050633934	6.4667371691	16.9146147331
H	6.0003909725	6.5080603524	17.2966449965
O	6.8478950644	6.0336605725	17.4802456197
H	6.5923968290	4.3063733958	17.1377130581
H	7.1682066303	2.8793129999	17.3318021083
O	6.4521918867	3.3571141365	16.8854418504
H	0.9045835666	2.4389083594	2.1823004673
H	1.5443458017	3.8118677182	2.5338299787
O	1.4949102070	2.8719920666	2.8174657597
H	4.3847633574	1.5193186601	2.1510590455
H	3.2727420893	2.1302251149	3.0178120173
O	4.1850725149	1.7648837915	3.0675280480
H	0.9002368453	6.1049629177	2.6382141095
H	2.2080988126	5.7935599085	3.3851149365
O	1.7680547489	5.6816015893	2.5221276135
H	3.4548801118	6.9335999955	2.1580498124
H	4.4210008446	8.0975250154	2.4764954884
O	4.1115712513	7.2390207800	2.8127982782
H	7.3310976961	6.5372041748	2.9188863629
H	5.8363820859	6.5651165209	2.5221966667
O	6.7428462630	6.5058526548	2.1482706745
H	6.8305333758	4.4243232022	2.4163248677
H	6.1705188901	3.1351202665	2.9699419596
O	7.0093861547	3.6322283821	2.9617333260
H	4.5901546917	4.4541234746	9.8744479380

Octagonal (Dissociation-penetration)

IS

C	3.4938223096	5.6717211546	9.4914931231

C	6.5473591587	7.1074450844	9.4914931231
C	6.5473591587	5.6715306889	9.4914931231
C	3.4938223096	7.1072542816	9.4914931231
C	4.2998625731	4.5181846202	9.4914932266
C	5.7411283091	4.5181846202	9.4914932266
C	8.0286530545	5.6715307914	9.4914931231
C	11.0821897832	7.1074450844	9.4914931231
C	11.0821896807	5.6717210517	9.4914931231
C	8.0286530545	7.1076364472	9.4914931231
C	8.8346107866	4.5181846202	9.4914932266
C	10.2764228713	4.5181846202	9.4914932266
C	12.5638659823	5.6717210517	9.4914931231
C	15.6172119088	7.1072542816	9.4914931231
C	15.6172119088	5.6717211546	9.4914931231
C	12.5638659823	7.1074450844	9.4914931231
C	13.3699060122	4.5181846202	9.4914932266
C	14.8114445389	4.5181846202	9.4914932266
C	3.4940135693	9.4145195923	9.4914931231
C	6.5471682355	10.8500516000	9.4914931231
C	6.5473590557	9.4143281271	9.4914931231
C	3.4940136729	10.8498602367	9.4914931231
C	4.2998625731	8.2607906957	9.4914932266
C	5.7411283091	8.2607906957	9.4914932266
C	8.0286530545	9.4143281271	9.4914931231
C	11.0827663594	10.9379642701	9.4880308534
C	11.0826842676	9.3530926034	9.4887727997
C	8.0288437543	10.8500516000	9.4914931231
C	8.8348842406	8.2607906957	9.4914931231
C	10.2762321715	8.2519721712	9.4910808330
C	12.5636747226	9.4143282301	9.4914931231
C	15.6172119088	10.8498602367	9.4914931231
C	15.6172119088	9.4145195923	9.4914931231
C	12.5638659823	10.8500516000	9.4916829439
C	13.3699060122	8.2607906957	9.4914931231
C	14.8112542786	8.2607906957	9.4914932266
C	3.4940136729	13.1573170316	9.4914931231
C	6.5473590557	14.5928490382	9.4914931231
C	6.5471682355	13.1571261079	9.4914931231
C	3.4940135693	14.5926576750	9.4914931231
C	4.2995885759	12.0035891339	9.4914932266
C	5.7411283091	12.0035891339	9.4914932266
C	8.0288438573	13.1571261079	9.4914931231
C	11.0819995408	14.5926577785	9.4914931231
C	11.0819995408	13.1571261079	9.4914931231

C	8.0288437543	14.5928490382	9.4914931231
C	8.8348842406	12.0035891339	9.4914931231
C	10.2763402375	12.0124076584	9.4919871679
C	12.5638660859	13.1571261079	9.4914931231
C	15.6172119088	14.5926576750	9.4914931231
C	15.6170205286	13.1573170316	9.4914931231
C	12.5638660859	14.5926577785	9.4914931231
C	13.3699059087	12.0035891339	9.4914931231
C	14.8114445389	12.0035891339	9.4914932266
H	6.1429058418	8.3251591636	17.0146146620
H	6.6796445529	9.7505850139	16.7331812375
O	6.6559103864	8.8401026509	16.3722505839
H	9.9702835645	8.2837518455	16.3373166959
H	8.4312625764	8.0495937967	16.3741280997
O	9.2879747564	7.5769859649	16.3664450842
H	6.1303880147	12.1363248747	16.3752390757
H	7.3158034580	11.5156126042	15.6148902945
O	6.9649325178	11.6560478440	16.5132071888
H	8.6235103822	12.8057429620	16.8260800328
H	9.4086512990	14.1406315018	16.7054546439
O	9.4302386275	13.2054114815	16.4429171428
H	12.5083635650	12.5759548333	16.0563569493
H	11.0661955303	12.4639951804	16.6274279090
O	11.9662164719	12.0572335836	16.6685972887
H	11.7380939589	10.2866611993	16.4011904225
H	12.1806973700	8.8438697337	16.7717151680
O	11.5544629875	9.3316927653	16.2153319484
H	6.1090100666	8.3805874109	1.8348363735
H	6.6878270475	9.7444603683	2.2758286426
O	6.5210601473	8.8309378067	2.5889603247
H	9.8816133660	8.2187255846	2.6873467755
H	8.3460589357	7.9635358911	2.7187279857
O	9.2104340454	7.5134951275	2.8062038230
H	6.0597865151	12.0128347962	2.5610681117
H	7.2836345111	11.5537112878	3.3689789020
O	6.9549915973	11.6472736942	2.4559670843
H	8.6390369488	12.7558541818	2.2546789444
H	9.6581979669	13.9171333796	2.2938106026
O	9.4314511418	13.0784517196	2.7309287627
H	12.4873146681	12.3656167543	3.0266110749
H	11.0408493327	12.2595753499	2.4764918002
O	11.9739530355	11.9864010062	2.2968701819
H	11.8432132955	10.1575317704	2.5116205912
H	12.2486119646	8.6557235937	2.3826634013

O	11.6158174852	9.2459743281	2.8182077084
H	10.3484210262	10.0797649538	13.1823523395

TS

C	3.3142145962	5.4920954614	9.3973162634
C	6.3677431915	6.9278287343	9.3973162634
C	6.3677431915	5.4919123848	9.3973162634
C	3.3142145962	6.9276407443	9.3973162634
C	4.1203025235	4.3385584652	9.3973123073
C	5.5614731633	4.3385584652	9.3973123073
C	7.8490480952	5.4919084692	9.3973162634
C	10.9025776660	6.9278287343	9.3973162634
C	10.9025815815	5.4920993972	9.3973162634
C	7.8490480952	6.9280167467	9.3973162634
C	8.6549444961	4.3385584652	9.3973123073
C	10.0968722154	4.3385584652	9.3973123073
C	12.3842605568	5.4920993972	9.3973162634
C	15.4376021173	6.9276407443	9.3973162634
C	15.4376021173	5.4920954614	9.3973162634
C	12.3842605568	6.9278287343	9.3973162634
C	13.1903396145	4.3385584652	9.3973123073
C	14.6318927491	4.3385584652	9.3973123073
C	3.3144065445	9.2349047449	9.3973162634
C	6.3675561770	10.6704499628	9.3973162634
C	6.3677471274	9.2347206480	9.3973162634
C	3.3144025884	10.6702619706	9.3973162634
C	4.1203025235	8.0811787160	9.3973123073
C	5.5614731633	8.0811787160	9.3973123073
C	7.8490480952	9.2347206480	9.3973162634
C	10.9038908654	10.8702880439	9.3894555708
C	10.9037003370	9.0956881328	9.3911400762
C	7.8492400210	10.6704499628	9.3973162634
C	8.6553230572	8.0811787160	9.3973162634
C	10.0966802895	8.0611572514	9.3963802053
C	12.3840686086	9.2347167122	9.3973162634
C	15.4376021173	10.6702619706	9.3973162634
C	15.4376021173	9.2349047449	9.3973162634
C	12.3842605568	10.6704499628	9.3975061934
C	13.1903396145	8.0811787160	9.3973162634
C	14.6317018212	8.0811787160	9.3973123073
C	3.3144025884	12.9777129675	9.3973162634
C	6.3677471274	14.4132582260	9.3973162634
C	6.3675561770	12.9775259733	9.3973162634
C	3.3144065445	14.4130702339	9.3973162634

C	4.1199269205	11.8239879792	9.3973123073
C	5.5614731633	11.8239879792	9.3973123073
C	7.8492360852	12.9775259733	9.3973162634
C	10.9023896780	14.4130662778	9.3973162634
C	10.9023896780	12.9775259733	9.3973162634
C	7.8492400210	14.4132582260	9.3973162634
C	8.6553230572	11.8239879792	9.3973162634
C	10.0966846046	11.8440094439	9.3984379365
C	12.3842566007	12.9775259733	9.3973162634
C	15.4376021173	14.4130702339	9.3973162634
C	15.4374111446	12.9777129675	9.3973162634
C	12.3842566007	14.4130662778	9.3973162634
C	13.1903435706	11.8239879792	9.3973162634
C	14.6318927491	11.8239879792	9.3973123073
H	5.9251242380	8.1342284945	17.0021821369
H	6.4549439689	9.5422105170	16.6299382732
O	6.3770524507	8.6241369047	16.2972944327
H	9.7512842384	8.0757017043	16.3006714340
H	8.2104003258	7.8369216641	16.2821908748
O	9.0692088575	7.3675461851	16.2939517325
H	5.9658906352	11.9117426987	16.2865840207
H	7.1483886820	11.3167500985	15.4994743433
O	6.8075134756	11.4389158119	16.4037492755
H	8.4463969759	12.6047609609	16.6690757490
H	9.2312740080	13.9314982290	16.4878505219
O	9.2192919582	12.9986222820	16.2159442012
H	12.3340778471	12.3105072763	16.1140231935
H	10.8590760390	12.2581753494	16.5910762994
O	11.7506802525	11.8581899211	16.7419396397
H	11.5194329654	10.1058726182	16.4484939729
H	12.0089344215	8.6521634314	16.6983056841
O	11.3389378491	9.1595557539	16.2160617866
H	5.9901156269	8.1763747229	1.7826461158
H	6.5533409541	9.5504637727	2.2204707255
O	6.4411787767	8.6223250765	2.5169758964
H	9.6869481968	8.0063794721	2.4682138945
H	8.1520199121	7.7717245273	2.5594400693
O	9.0094406937	7.2980273639	2.5205069565
H	5.8485623617	11.8098057193	2.4788135226
H	7.0906135930	11.4019361256	3.2881026600
O	6.7417215471	11.4380867866	2.3787751531
H	8.4538015783	12.5326011177	2.0927326779
H	9.4366910809	13.7234827518	2.1482155049
O	9.2275257342	12.8790720883	2.5821226768

H	12.3080214476	12.2042144605	2.9715094486
H	10.8862277020	12.0986716411	2.3681015736
O	11.8200717561	11.7999075291	2.2363819611
H	11.6199759463	10.0173280761	2.3589073827
H	12.0528329680	8.5167591781	2.3147821607
O	11.3338656138	9.1005360154	2.5969837563
H	10.0912406057	9.9669636295	9.3917425866

Hexagonal (Adsorption-penetration)

IS

C	3.8355900000	6.0112810000	9.1776090000
C	6.9154930000	7.4617400000	9.1093790000
C	6.9156920000	5.9952610000	9.0615180000
C	3.8375680000	7.4449290000	9.2258660000
C	4.6434890000	4.8549070000	9.1471530000
C	6.0840600000	4.8475900000	9.1020610000
C	8.3530990000	5.9835920000	9.0682420000
C	11.4096650000	7.4425560000	9.2078690000
C	11.4102580000	6.0089070000	9.1918500000
C	8.3586360000	7.4743970000	9.1062150000
C	9.1617880000	4.8479850000	9.1410220000
C	10.6015690000	4.8507540000	9.1890810000
C	12.8947350000	6.0087090000	9.2110340000
C	15.9540690000	7.4453250000	9.2375350000
C	15.9522900000	6.0118730000	9.2001560000
C	12.8967130000	7.4421610000	9.2276470000
C	13.7061930000	4.8543140000	9.2149890000
C	15.1370740000	4.8590600000	9.2029250000
C	3.8375680000	9.7584670000	9.3235650000
C	6.8452850000	11.1851930000	9.9358680000
C	6.9107470000	9.7305800000	9.4529090000
C	3.8338100000	11.1879630000	9.3296970000
C	4.6474440000	8.6007090000	9.2770890000
C	6.0911800000	8.6013020000	9.2602790000
C	8.3307500000	9.7699370000	9.3771620000
C	11.4051160000	11.1784700000	9.3196110000
C	11.4070940000	9.7537200000	9.2911310000
C	8.3529010000	11.1755020000	9.6012370000
C	9.1645570000	8.5977420000	9.2082650000
C	10.5986010000	8.5959630000	9.2331840000
C	12.9004700000	9.7539180000	9.2840110000
C	15.9457640000	11.1828200000	9.2982510000
C	15.9511040000	9.7564890000	9.3033920000

C	12.8988880000	11.1806450000	9.2940980000
C	13.7095560000	8.5955670000	9.2606740000
C	15.1416230000	8.5963570000	9.2701670000
C	3.8231310000	13.4993240000	9.2236910000
C	6.8955190000	14.9256550000	9.1501200000
C	6.8672370000	13.4581880000	9.3031950000
C	3.8286680000	14.9274360000	9.1797860000
C	4.6347870000	12.3482900000	9.3184240000
C	6.0615140000	12.3631230000	9.4155290000
C	8.3378700000	13.4755920000	9.3690530000
C	11.4082800000	14.9240730000	9.2312060000
C	11.4029400000	13.4916110000	9.2727380000
C	8.3384640000	14.9131960000	9.1922450000
C	9.1582290000	12.3320730000	9.4610180000
C	10.5964270000	12.3366220000	9.3413650000
C	12.8937460000	13.4927980000	9.2533570000
C	15.9457640000	14.9296110000	9.1948170000
C	15.9416100000	13.4977420000	9.2230980000
C	12.8961190000	14.9226890000	9.2274480000
C	13.7048090000	12.3378090000	9.2687830000
C	15.1354910000	12.3397870000	9.2618610000
H	4.1688360000	8.8999390000	17.7994800000
H	4.6041330000	10.3591000000	17.5455420000
O	4.4963460000	9.4924630000	17.1047080000
H	7.9395580000	8.9084420000	17.1952860000
H	6.4093950000	8.6887180000	17.0764260000
O	7.2445860000	8.2168340000	17.2500690000
H	4.1652760000	12.8011890000	17.2263370000
H	5.4157890000	12.1714830000	16.6017720000
O	4.9994790000	12.3613430000	17.4636630000
H	6.6599720000	13.5936610000	17.5287300000
H	7.4544230000	14.8188580000	17.0074040000
O	7.4126930000	13.8489860000	16.9585530000
H	10.4910140000	13.0209130000	16.7597920000
H	9.0601340000	13.0729280000	17.3438130000
O	9.9453610000	12.6530570000	17.4721680000
H	9.6769850000	10.9134540000	17.2449280000
H	10.2186820000	9.4550840000	17.3418370000
O	9.4649720000	9.9722590000	17.0224340000
H	4.1644850000	8.9928910000	1.9777200000
H	4.6591130000	10.3191500000	2.5933840000
O	4.4921930000	9.3763700000	2.8055930000
H	7.7471250000	8.7193720000	2.7294510000
H	6.2523640000	8.4294390000	2.9560980000

O	7.1186060000	7.9834630000	2.8787690000
H	3.8834510000	12.5472490000	2.6370920000
H	5.1507740000	12.3508620000	3.4857320000
O	4.7918180000	12.2039180000	2.5908130000
H	6.5053140000	13.3956920000	2.1472110000
H	7.3130150000	14.6901090000	2.4179600000
O	7.2647600000	13.7471320000	2.6511340000
H	10.3547490000	13.0519640000	2.7802790000
H	8.9027070000	13.0149800000	2.2607320000
O	9.8110740000	12.6765920000	2.0700800000
H	9.5440820000	10.8924900000	2.4157850000
H	10.0426650000	9.4620070000	2.7939250000
O	9.2446550000	10.0066700000	2.7343960000
H	6.6997240000	11.1664060000	11.0317220000

TS

C	3.8637448465	6.0237714299	9.7481132910
C	6.9032010696	7.4123707714	9.7378000270
C	6.9087525381	5.9698821388	9.7301412308
C	3.8611623709	7.4500849741	9.7554401214
C	4.6613461207	4.8509420229	9.7433423847
C	6.0993274177	4.8200296494	9.7364442918
C	8.3836318303	5.9701090727	9.7310354332
C	11.4326638724	7.4498924609	9.7523146970
C	11.4294903948	6.0231819202	9.7502161037
C	8.3899628282	7.4123388420	9.7371991602
C	9.1935118272	4.8206241514	9.7421696013
C	10.6315073343	4.8507407802	9.7496689912
C	12.9169408770	6.0289328897	9.7533116245
C	15.9782598719	7.4502814721	9.7571531622
C	15.9814353389	6.0295194039	9.7516616180
C	12.9203063684	7.4500919568	9.7554881050
C	13.7361323368	4.8812656323	9.7540282712
C	15.1618583653	4.8816638785	9.7522360391
C	3.8607653719	9.7596559919	9.7696272254
C	6.6103470082	11.0878420342	9.8633273174
C	6.9044020341	9.7416839410	9.7907238637
C	3.8179490889	11.1897464878	9.7704437360
C	4.6750033480	8.6011059990	9.7631423765
C	6.1064464151	8.5582777373	9.7611242973
C	8.3900463950	9.7415846565	9.7790411443
C	11.4759123325	11.1901684370	9.7687263658
C	11.4332527036	9.7602624629	9.7642011836
C	8.6830216881	11.0880649808	9.8151383921

C	9.1877543719	8.5578897191	9.7527635762
C	10.6194203882	8.6011179715	9.7560282314
C	12.9339783175	9.7529265051	9.7630577375
C	15.9552799451	11.1800440147	9.7651339797
C	15.9649829238	9.7521260214	9.7663756954
C	12.9441069718	11.1808424970	9.7643791755
C	13.7329508689	8.6003249726	9.7601979050
C	15.1654158704	8.6001254827	9.7618253018
C	3.7957691489	13.5167709019	9.7545596079
C	6.9028544583	14.8808472495	9.7445250246
C	6.8811037065	13.4526350270	9.7693375241
C	3.8260905142	14.9545993516	9.7480278083
C	4.5824432867	12.3732738673	9.7701436272
C	5.9931108761	12.3577695290	9.7880073047
C	8.4128165893	13.4529891240	9.7783758444
C	11.4661756839	14.9540133394	9.7557823121
C	11.4975159831	13.5163953653	9.7618047239
C	8.3906082113	14.8812605203	9.7504530658
C	9.3004019386	12.3578478577	9.7932123108
C	10.7108288713	12.3731048023	9.7731755828
C	12.9516406813	13.5138148851	9.7584726104
C	15.9552799451	14.9571713506	9.7501823096
C	15.9473604700	13.5139999100	9.7539006425
C	12.9425147464	14.9567908109	9.7552768209
C	13.7404988068	12.3473249451	9.7606788609
C	15.1592848729	12.3469249614	9.7597587852
H	4.1970758560	8.8597435915	17.7923248021
H	4.6347783277	10.3247364409	17.5664359254
O	4.5383133959	9.4635812146	17.1130252519
H	7.9906938608	8.8834366361	17.2033068576
H	6.4473794280	8.6727591839	17.1015337101
O	7.2859640906	8.1962289834	17.2567073731
H	4.1956875484	12.7740435742	17.2770476292
H	5.4189795825	12.1404782482	16.5977624538
O	5.0397229520	12.3340441768	17.4750932271
H	6.7021977951	13.5353923556	17.5288095811
H	7.4752509487	14.7923041783	17.0518159907
O	7.4474123736	13.8251174096	16.9641447675
H	10.5337682814	12.9747755758	16.7792120960
H	9.0914570838	13.0595426589	17.3421558325
O	9.9666652841	12.6239189683	17.4827012065
H	9.6762470574	10.8635161642	17.2540412574
H	10.2342367241	9.4236557181	17.4378808901
O	9.5027214306	9.9122802344	17.0317507996

H	4.6219136836	9.0013047886	1.9854807583
H	5.1036500748	10.3307352306	2.6029648075
O	4.9583734351	9.3839171798	2.8117295031
H	8.2318484543	8.7881564828	2.8092663141
H	6.7106282332	8.5123057775	2.9581931474
O	7.5832411987	8.0658247574	2.9639076455
H	4.3483117912	12.6329311046	2.6039050743
H	5.5825747788	12.4161430102	3.5006622236
O	5.2523278780	12.2757504993	2.5952331928
H	6.9838600157	13.4264144606	2.1659768924
H	7.7124546678	14.7676991045	2.4288581118
O	7.7248372269	13.8246884582	2.6641871246
H	10.8017669769	13.0819443991	2.7367724816
H	9.3421277000	13.0973801118	2.2376631540
O	10.2143356828	12.6563792274	2.0911652387
H	9.9170107701	10.9341735695	2.4809387487
H	10.5198187874	9.5215074946	2.7036117197
O	9.7145845654	10.0379396617	2.8561926446
H	7.6553876823	11.4239901754	9.1675271758

Octagonal (Adsorption-penetration)

IS

C	0.7429754474	1.7478953427	9.4142546244
C	3.8126094889	3.1972371299	9.5800267115
C	3.8125933974	1.7600265960	9.4016031998
C	0.7478328911	3.1810911961	9.4572958705
C	1.5499791108	0.5938596312	9.3783480014
C	2.9898168247	0.5975651876	9.3425277739
C	5.2555837394	1.7474623655	9.3611676800
C	8.3280877493	3.1736350903	9.4150000442
C	8.3221950866	1.7447701435	9.3666131160
C	5.2839039421	3.2143420102	9.5168220788
C	6.0668363733	0.5978964169	9.3053737756
C	7.5077585270	0.5902443041	9.3387466951
C	9.8103159738	1.7428147558	9.3687285394
C	12.8632958624	3.1797093800	9.4273139648
C	12.8604376556	1.7493853296	9.3991147893
C	9.8136621134	3.1751129847	9.4008795357
C	10.6194178726	0.5859954657	9.3685177842
C	12.0504873270	0.5903234290	9.3798860692
C	0.7453662574	5.4938601378	9.5030330507
C	3.8205031420	6.9033474542	9.5791804228
C	3.7973465306	5.4976847129	9.8050171932

C	0.7440736379	6.9195044813	9.4717597047
C	1.5542028597	4.3359492878	9.5331981194
C	2.9916136961	4.3411603948	9.6639880508
C	5.3055451636	5.4886995186	10.1415874330
C	8.3139128949	6.9150814548	9.5115787825
C	8.3182378837	5.4847864163	9.5220177294
C	5.2401008739	6.9427666416	9.6563776606
C	6.0900755382	4.3096207902	9.6244341411
C	7.5166026695	4.3244076098	9.5184842209
C	9.8101972559	5.4896871555	9.4771375944
C	12.8563149790	6.9190243658	9.4548996350
C	12.8582178561	5.4917880838	9.4668447947
C	9.8054272042	6.9168149508	9.4785759414
C	10.6208518923	4.3330331559	9.4358127516
C	12.0513342794	4.3348682106	9.4401116455
C	0.7423446480	9.2307733889	9.3858398700
C	3.7986223882	10.6900394173	9.2708268295
C	3.7925528769	9.1987961377	9.3070910652
C	0.7416868885	10.6628481620	9.3705902940
C	1.5530949017	8.0765160312	9.4203003000
C	2.9870160615	8.0752814957	9.4062358671
C	5.2356440484	9.2113472478	9.3108792840
C	8.3150735834	10.6606281716	9.3581557859
C	8.3131383880	9.2282888144	9.4061319989
C	5.2355095068	10.6779978829	9.2649020737
C	6.0597943032	8.0711283715	9.4603014745
C	7.5036510675	8.0716811816	9.4679887746
C	9.8029619966	9.2279645697	9.4059847854
C	12.8611030671	10.6631336372	9.3779879785
C	12.8592295955	9.2307560604	9.3940754041
C	9.8048087374	10.6600710703	9.3679473415
C	10.6149321136	8.0755337548	9.4368268379
C	12.0470063070	8.0764961541	9.4255080066
H	3.3091429056	4.0640369964	16.6954876596
H	3.3267551568	4.2116410818	18.2368709107
O	3.6197689867	4.6385664541	17.4143087280
H	7.0461683837	3.8994875899	16.8933861136
H	5.5593492242	3.7891718107	17.2300162944
O	6.3170557456	3.2422044181	16.9613232235
H	3.4865914921	7.7940622851	16.8779665812
H	4.2061947676	6.5384716003	17.3991580735
O	4.2500068205	7.5176376960	17.4091606325
H	5.7451628250	8.5275759183	17.0850793455
H	6.3101094281	9.9684246256	17.3061476097

O	6.5133398187	9.1177060479	16.8875482674
H	9.4766534206	8.2255680543	16.5913994790
H	8.0600984079	8.3965547825	17.2051686268
O	8.9261746160	7.9226964133	17.3297242371
H	8.6048274463	6.1607078866	17.1605371856
H	8.8737378644	4.8649179536	17.9557953513
O	8.5032871749	5.1739536693	17.1148338529
H	4.6786054996	4.0325778081	2.8804797807
H	4.1305358474	5.3791796034	2.4195905554
O	3.8313381066	4.4867860072	2.6648793258
H	7.0974761834	4.1872843471	3.1441306475
H	6.5704485906	3.1648075119	4.1786263589
O	6.4507551100	3.4452287457	3.2585008009
H	3.6200655494	7.7152888564	1.8262396057
H	3.7248274913	7.3849791733	3.3328006850
O	4.2490025115	7.4410447895	2.5148345330
H	5.7993651119	8.4201646897	2.6346164676
H	6.3321970701	9.8578260381	2.3799791935
O	6.5911397795	8.9959673565	2.7414362043
H	9.6544409512	8.3371801536	2.6612448356
H	8.1479809806	8.3778980168	2.2693657349
O	9.0051804447	7.9144091371	2.0778779695
H	8.6693591527	6.2560408112	2.6371163019
H	9.2143141494	4.8099790099	2.4924833350
O	8.5418220990	5.3239683462	2.9646106280
H	5.4495265411	5.5110003266	11.2375907561

TS

C	0.7379662270	1.7384092319	10.0009719598
C	3.7941584750	3.1677648615	10.0006987158
C	3.7789108985	1.7170078799	10.0009954710
C	0.7505033653	3.1695652715	10.0009016357
C	1.5380345346	0.5769466637	10.0010308670
C	2.9651907890	0.5783824245	10.0010926992
C	5.2408098330	1.7034852270	10.0010640479
C	8.3451079966	3.1585948404	10.0009725194
C	8.3407510857	1.7297137223	10.0010530522
C	5.2619857554	3.1185337322	10.0008043860
C	6.0854813516	0.5784288688	10.0011569362
C	7.5207746900	0.5791440679	10.0010995872
C	9.8212632411	1.7326544609	10.0010484848
C	12.8646317762	3.1721697117	10.0009497559
C	12.8655909927	1.7388351951	10.0009953190
C	9.8321876714	3.1663975302	10.0009957677

C	10.6318447732	0.5773538953	10.0010480579
C	12.0596881608	0.5765005261	10.0010282862
C	0.7578982977	5.4798021907	10.0008246774
C	3.8346597340	6.9053683470	10.0006938590
C	3.8297799745	5.4784401205	10.0003154061
C	0.7579146456	6.9021424484	10.0008770069
C	1.5642906563	4.3212464241	10.0007758877
C	2.9999572159	4.3161573471	10.0005586189
C	5.2489085033	5.3062910625	9.9997229927
C	8.3118711825	6.9012036846	10.0008091855
C	8.3105530261	5.4840328320	10.0007913375
C	5.2468365949	7.0746256210	10.0005375769
C	6.1089639044	4.2258970081	10.0006081762
C	7.5343905997	4.2901918791	10.0007951558
C	9.8206092812	5.4864806618	10.0008691752
C	12.8714172374	6.9026254473	10.0009038943
C	12.8681111302	5.4797611929	10.0008842676
C	9.8214151452	6.8984713475	10.0008664225
C	10.6260573445	4.3209922926	10.0009371292
C	12.0597201933	4.3226536535	10.0009290061
C	0.7492722861	9.2120891683	10.0010186301
C	3.7854022375	10.6768702479	10.0012165348
C	3.8047796144	9.2158345638	10.0011564472
C	0.7370082645	10.6528222617	10.0010427897
C	1.5669680822	8.0654556743	10.0009638777
C	3.0027518354	8.0710162269	10.0009892450
C	5.2574085694	9.2620461359	10.0011476634
C	8.3435311724	10.6566410328	10.0010662780
C	8.3496637014	9.2251504551	10.0009858261
C	5.2394457222	10.6932360139	10.0012257532
C	6.1080416330	8.1628867430	10.0008830406
C	7.5341186263	8.0958793970	10.0008791826
C	9.8325569816	9.2169854019	10.0009873603
C	12.8688140371	10.6500740934	10.0010326791
C	12.8719270883	9.2095213740	10.0010063769
C	9.8203324388	10.6554749234	10.0010502909
C	10.6289037102	8.0643484091	10.0009349122
C	12.0618086498	8.0624836590	10.0009534078
H	3.2963356924	4.0544559214	16.7087938285
H	3.3155606744	4.2287553490	18.2447572633
O	3.6201532764	4.6359386283	17.4154321908
H	7.0482761750	3.9130310139	16.9139626229
H	5.5593586158	3.7902982588	17.2538142033
O	6.3163355270	3.2553535030	16.9573082198

H	3.4773706690	7.7958923432	16.9018061056
H	4.1917086272	6.5298595617	17.4064939837
O	4.2536389970	7.5088610307	17.4068996597
H	5.7505828593	8.5264060808	17.1007784649
H	6.3251348799	9.9600587979	17.3346068727
O	6.5112802142	9.1205036684	16.8852961343
H	9.4856031112	8.2389490491	16.6084220261
H	8.0595951490	8.3994402655	17.2097727746
O	8.9232738679	7.9202302969	17.3300700865
H	8.6007753092	6.1677596112	17.1676417280
H	8.8755743146	4.8672199454	17.9516539834
O	8.5026247405	5.1802263157	17.1126865007
H	4.6826449069	4.0320344465	2.8641899305
H	4.1281153202	5.3752628743	2.4000766942
O	3.8316118321	4.4881229707	2.6684343103
H	7.1122018287	4.1796257819	3.1255267486
H	6.5825569593	3.1758473351	4.1771366394
O	6.4525268996	3.4501709852	3.2575663630
H	3.6258246648	7.7192842583	1.8184519951
H	3.7142665712	7.3765300487	3.3212638779
O	4.2490094983	7.4417601004	2.5118265990
H	5.7986437926	8.4324873967	2.6231337793
H	6.3421761147	9.8633565226	2.3564970727
O	6.5885066131	9.0089011154	2.7443030921
H	9.6466166058	8.3350806350	2.6331997214
H	8.1377607910	8.3580944572	2.2609002170
O	8.9909379519	7.8794366136	2.0818728771
H	8.6727776165	6.2498767083	2.6329982302
H	9.2182073228	4.8069538511	2.4646830945
O	8.5645293208	5.3159381966	2.9678632626
H	6.0742188643	6.1919781132	10.0041533429

References

1. Q. Zhang, M. Ju, L. Chen, X. C. Zeng, *J. Phys. Chem. Lett.* 2016, **7**, 3395-3400.
2. J. M. H. Kroes, A. Fasolino, M. I. Katsnelson, *Phys. Chem. Chem. Phys.* 2017, **19**, 5813-5817.
